# Re-Evaluating *Botryosphaeriales*: Ancestral State Reconstructions of Selected Characters and Evolution of Nutritional Modes

**DOI:** 10.3390/jof9020184

**Published:** 2023-01-29

**Authors:** Achala R. Rathnayaka, K. W. Thilini Chethana, Alan J. L. Phillips, Jian-Kui Liu, Milan C. Samarakoon, E. B. Gareth Jones, Samantha C. Karunarathna, Chang-Lin Zhao

**Affiliations:** 1 School of Science, Mae Fah Luang University, Chiang Rai 57100, Thailand; 2 Center of Excellence in Fungal Research, Mae Fah Luang University, Chiang Rai 57100, Thailand; 3 Department of Plant Medicine, National Chiayi University, 300 Syuefu Road, Chiayi City 60004, Taiwan; 4 Faculdade de Ciências, Biosystems and Integrative Sciences Institute (BioISI), Universidade de Lisboa, Campo Grande, 1749-016 Lisbon, Portugal; 5 School of Life Science and Technology, University of Electronic Science and Technology of China, Chengdu 610054, China; 6 Department of Entomology and Plant Pathology, Faculty of Agriculture, Chiang Mai University, Chiang Mai 50200, Thailand; 7 Department of Botany and Microbiology, College of Science, King Saud University, P.O. Box 2455, Riyadh 11451, Saudi Arabia; 8 Center for Yunnan Plateau Biological Resources Protection and Utilization, College of Biological Resource and Food Engineering, Qujing Normal University, Qujing 655011, China; 9 Key Laboratory for Forest Resources Conservation and Utilization in the Southwest Mountains of China, Ministry of Education, Southwest Forestry University, Kunming 650224, China

**Keywords:** ancestral characters, BEAST, divergence times, morphology, phylogeny

## Abstract

*Botryosphaeriales* (*Dothideomycetes*, *Ascomycota*) occur in a wide range of habitats as endophytes, saprobes, and pathogens. The order *Botryosphaeriales* has not been subjected to evaluation since 2019 by Phillips and co-authors using phylogenetic and evolutionary analyses. Subsequently, many studies introduced novel taxa into the order and revised several families separately. In addition, no ancestral character studies have been conducted for this order. Therefore, in this study, we re-evaluated the character evolution and taxonomic placements of *Botryosphaeriales* species based on ancestral character evolution, divergence time estimation, and phylogenetic relationships, including all the novel taxa that have been introduced so far. Maximum likelihood, maximum parsimony, and Bayesian inference analyses were conducted on a combined LSU and ITS sequence alignment. Ancestral state reconstruction was carried out for conidial colour, septation, and nutritional mode. Divergence times estimates revealed that *Botryosphaeriales* originated around 109 Mya in the early epoch of the Cretaceous period. All six families in *Botryosphaeriales* evolved in the late epoch of the Cretaceous period (66–100 Mya), during which Angiosperms also appeared, rapidly diversified and became dominant on land. Families of *Botryosphaeriales* diversified during the Paleogene and Neogene periods in the Cenozoic era. The order comprises the families *Aplosporellaceae*, *Botryosphaeriaceae*, *Melanopsaceae*, *Phyllostictaceae*, *Planistromellaceae* and *Saccharataceae*. Furthermore, current study assessed two hypotheses; the first one being “All *Botryosphaeriales* species originated as endophytes and then switched into saprobes when their hosts died or into pathogens when their hosts were under stress”; the second hypothesis states that “There is a link between the conidial colour and nutritional mode in botryosphaerialean taxa”. Ancestral state reconstruction and nutritional mode analyses revealed a pathogenic/saprobic nutritional mode as the ancestral character. However, we could not provide strong evidence for the first hypothesis mainly due to the significantly low number of studies reporting the endophytic botryosphaerialean taxa. Results also showed that hyaline and aseptate conidia were ancestral characters in *Botryosphaeriales* and supported the relationship between conidial pigmentation and the pathogenicity of *Botryosphaeriales* species.

## 1. Introduction

### 1.1. Botryosphaeriales

*Botryosphaeriales* was introduced to accommodate *Botryosphaeriaceae* by Schoch et al. [1]. Following consecutive studies, the families *Planistromellaceae* [2], *Phyllostictaceae* [3], *Aplosporellaceae*, *Melanopsaceae*, *Saccharataceae* [4], *Septorioideaceae* [5], *Endomelanconiopsisaceae*, and *Pseudofusicoccumaceae* [6] were recognized in *Botryosphaeriales*. In a revision based on morphology and phylogeny, Phillips et al. [7] synonymized *Endomelanconiopsisaceae* under *Botryosphaeriaceae*. *Pseudofusicoccumaceae* and *Septorioideaceae* were also synonymized under *Phyllostictaceae* and *Saccharataceae*, respectively. Currently, six families are accepted in *Botryosphaeriales*, i.e., *Aplosporellaceae*, *Botryosphaeriaceae*, *Melanopsaceae*, *Phyllostictaceae*, *Planistromellaceae,* and *Saccharataceae* [7,8,9].

*Botryosphaeriales* is an order with a variety of lifestyles ranging from endophytes to pathogens and saprobes [10] on a wide range of monocotyledonous and dicotyledonous hosts [11] and lichens [12,13]. Most of the taxa in *Botryosphaeriales* are endophytes living in the healthy tissues of woody plants for extended periods [10]. Species of *Botryosphaeria*, *Diplodia*, *Dothiorella*, *Lasiodiplodia*, *Neofusicoccum*, *Phyllosticta*, *Pseudofusicoccum* and *Saccharata* include endophytes [8,10,14,15]. Some *Botryosphaeriales* species are important phytopathogens associated with canker diseases, with a worldwide distribution and a broad host range, causing severe ecological and economical damage [7]. Pathogenic species in *Botryosphaeriales*, such as quiescence pathogens (such as *Botryosphaeria* and *Lasiodiplodia* species) cause diseases following an initial stress factor, such as drought or infection by another weak pathogen [5,16]. As an example, water stress affects disease development of *Lasiodiplodia theobromae* and *Sphaeropsis sapinea* on *Platanus occidentalis* and *Pinus resinosa*, respectively [17]. 

### 1.2. Previous Revisions for the Families in Botryosphaeriales

Theissen and Sydow [18] introduced *Botryosphaeriaceae* to accommodate *Botryosphaeria*, *Dibotryon* and *Phaeobotryon* [7,19]. *Botryosphaeriaceae* species have a range of nutritional modes from saprobic to parasitic or endophytic [10,20,21,22,23,24,25,26,27]. Members of this family are cosmopolitan in distribution and occur on a wide range of monocotyledonous and dicotyledonous hosts: on woody branches, leaves, stems and culms of grasses, and on twigs and in the thalli of lichens [12,21,28,29,30]. Liu et al. [11] accepted 29 genera in *Botryosphaeriaceae* based on morphology and molecular data. Phillips et al. [19] provided detailed descriptions and keys for 17 genera in *Botryosphaeriaceae*. Burgess et al. [31] and Garcia et al. [32] included 24 genera in *Botryosphaeriaceae* based on morpho-molecular data. However, Dissanayake et al. [33] mentioned that this family consists of 22 genera. This is the largest family in *Botryosphaeriales* [8,34]. Nearly 280 species have been described in *Botryosphaeriaceae* based on DNA sequence data [35].

*Aplosporellaceae* was introduced by Slippers et al. [4] to accommodate *Aplosporella* and *Bagnisiella*. *Aplosporella* are asexual morphs, while *Bagnisiella* species are known through their sexual morphs [36]. Sharma et al. [37] introduced *Alanomyces* in this family, which currently consist of two genera: *Aplosporella* and *Alanomyces* [34]. *Melanopsaceae* was introduced with *Melanops* as the type genus [4] and remains the only genus in the family [34].

Wikee et al. [3] reinstated *Phyllostictaceae* as a separate family in *Botryosphaeriales* to accommodate *Phyllosticta*, which consists of *Phyllosticta* and *Pseudofusicoccum* [34]. *Phyllosticta* species are mostly endophytes, but several are plant pathogens that cause leaf spots in a broad range of hosts worldwide [38,39,40,41,42]. Barr [43] introduced *Planistromellaceae*, which currently comprises two genera, namely, *Kellermania* and *Umthunziomyces* [33]. *Saccharataceae* is another family in *Botryosphaeriales* introduced by Slippers et al. [4] and consists of *Pileospora*, *Saccharata* and *Septorioides* [33].

### 1.3. Morphologies of Botryosphaerialean Taxa

**Figure 1:**
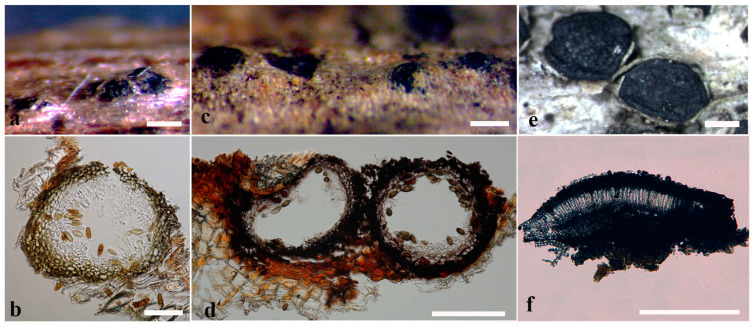
Uni-loculate and multi-loculate ascostromata/conidiomata. (**a**,**b**) Uni-loculate conidiomata of *Dothiorella viticola*. (**c**,**d**) Uni-loculate ascostromata of *Sphaeropsis* sp. (**e**,**f**) Multi-loculate ascostromata of *Aplosporella thailandica* [36]. Scale bars: (**a**,**c**,**d**) = 200 μm, b = 100 μm, (**e**,**f**) = 500 μm.

Morphological characters vary between families in this order. Uni-loculate and multi-loculate ascostromata can be found in *Botryosphaeriales* (Figure 1). *Aplosporellaceae*, *Melanopsaceae* and *Planistromellaceae* are characterized by multiloculate ascostromata, while *Botryosphaeriaceae*, *Phyllostictaceae* and *Saccharataceae* have uni-loculate ascostromata [7]. In *Saccharataceae* and *Phyllostictaceae*, solitary, uni-loculate ascostromata have been recorded. In *Botryosphaeriaceae*, uni-loculate ascostromata are mostly solitary, but in some genera, such as *Botryosphaeria*, *Diplodia* and *Neofusicoccum*, they can be aggregated, which give the impression of being multi-loculate [7].

**Figure 2:**
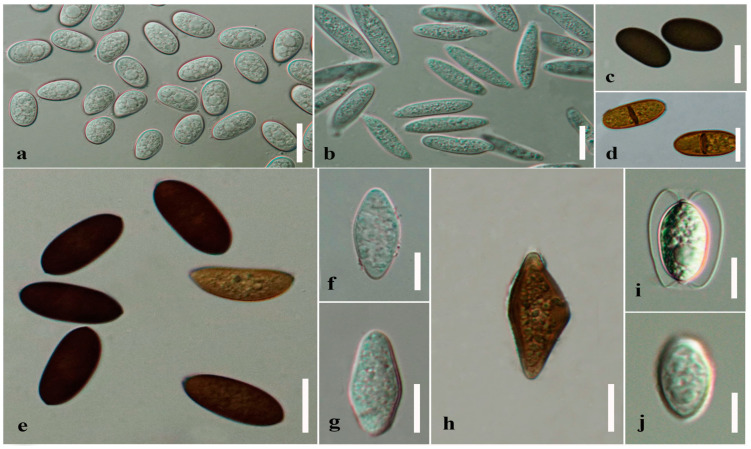
Conidial and ascospore colour and septation. (**a**) Hyaline and aseptate conidia of *Lasiodiplodia* sp. (**b**) Hyaline and aseptate conidia of *Botryosphaeria dothidea*. (**c**) Brown and aseptate conidia of *Aplosporella thailandica* [36]. (**d**) Brown and septate conidia of *Dothiorella viticola*. (**e**) Brown and aseptate ascospores of *Sphaeropsis* sp. (**f**) Hyaline and aseptate ascospore of *Botryosphaeria fabicerciana*. (**g**) Hyaline and aseptate ascospore of *Neofusicoccum parvum*. (**h**) Brown and aseptate ascospore of *Barriopsis archontophoenicis*. (**i**) Wing-like appendages of *Neodeightonia palmicola* ascospore (in water). (**j**) *Neodeightonia palmicola* ascospore in 100% lactic acid. Scale bars: (**a**,**d**,**e**) = 20 μm, (**b**,**c**,**f**–**j**) = 10 μm.

Ascospores and conidia in *Botryosphaeriales* have a wide range of morphologies, such as pigmented or hyaline, septate or aseptate and the presence or absence of a mucilaginous sheath (Table 1, Figure 2). *Botryosphaeriaceae* species have a wide range of conidial morphologies, such as fusiform to ovoid or elliptical, fusicoccum-like, hyaline, aseptate and thin-walled. Hyaline and, aseptate conidia become one or two septate and some species become pale brown before germination (*Diplodia corticola*, *D*. *cupressi* and *D*. *mutila*) [19]. Thick-walled and hyaline or brown diplodia-like conidia also occur in *Botryosphaeriaceae*. They can be aseptate, one-septate or even two- or multi-septate and have ovoid conidia with broadly rounded ends [19]. In *Diplodia* and *Lasiodiplodia*, conidia can remain hyaline for a long time and become brown and one-septate only after they are discharged from the conidiomata [19].

The mucilaginous sheath is one morphological character used to separate the families in *Botryosphaeriales*. Ascospores with mucilaginous sheath and gelatinous caps have been recorded in *Melanopsaceae* and *Phyllostictaceae*, respectively [7]. Mature ascospores of some species such as *Botryosphaeria agaves* and *Melanops tulasnei*, and immature ascospores of *Phaeobotryon cercidis,* have a mucilaginous sheath [4,7,11]. *Neodeightonia palmicola* has wing-like appendages when mounted in water. However, these wing-like appendages are not observed when mounted in 100% lactic acid (Figure 2i,j). Phillips et al. [7] suggested that these wing-like structures are a type of membrane surrounding the ascospores that enlarge and swell when water is absorbed [7]. 

Spore morphology influences survival in the environment [44]. Spore wall thickness and pigmentation protect spores from extreme conditions, such as heat, microbial attack and UV radiation [44,45]. Pigmentation of conidia is due to the melaninization of the conidial wall or the deposition of oxidized polymers of phenolic compounds [46]. Mainly three pigments (carotenoids, melanin and mycosporines) occur in fungi, and they act as antioxidants and reduce the damage from UV exposure [45]. Melanin can be found in pathogenic, as well as in saprobic taxa, and contributes to survival under harsh environmental conditions [47]. However, melanin production has more of an impact on pathogens because it is directly linked with virulence and pathogenicity [47,48].

### 1.4. Ancestral State Reconstructions for Fungi

There have been relatively few studies on ancestral state reconstructions in fungi to determine character evolution [4,51,52,53,54]. For more than two decades, character evolution has been highly contentious in lichen systematics [51]. Ekman et al. [51] studied the evolution of the ascus in *Lecanorales* using ancestral state reconstruction. Slippers et al. [4] performed ancestral state reconstructions for selected characters in *Botryosphaeriales*, such as ascospore colour, the presence or absence of ascospore septa, conidial colour, the presence or absence of conidial septa and presence or absence of a mucus sheath. However, they did not consider all the species of *Botryosphaeriales*. No studies have been conducted using ancestral state reconstruction or nutritional mode evolution with all the families or genera in *Botryosphaeriales*.

### 1.5. Objectives of the Current Study

This study aims to provide an updated phylogenetic tree for *Botryosphaeriales* using LSU and ITS sequence data (for ordinal level). Divergence time estimates were performed using the updated phylogeny of *Botryosphaeriales*. Furthermore, ancestral state reconstruction was performed for selected characters, i.e., conidial colour and septation and nutritional mode in *Botryosphaeriales*. Two hypotheses were assessed in the current study. The first hypothesis assessed was that “All *Botryosphaeriales* species originated as endophytes and then switched into saprobes when their hosts died or into pathogens when their hosts were under stress”. The second hypothesis tested was that “There is a link between conidial colour and nutritional mode in botryosphaerialean taxa”. Both hypotheses were tested based on the results from ancestral state reconstructions.

## 2. Materials and Methods

### 2.1. Data Collection and Analyses

Sequences were obtained from the GenBank for taxa reported in the recently published data on *Botryosphaeriales* species (Table S1) [7,8,11]. All the reported nutritional modes of each *Botryosphaeriales* species were considered for the ancestral state reconstruction analysis of nutritional mode. For other analyses, i.e., maximum likelihood (ML), maximum parsimony (MP) and Bayesian inference (BI) and character analysis, one or two strains of each taxon were used. The tree file resulting from the evolution analysis was used for ancestral state reconstructions. Sequences of each locus were aligned with MAFFT v. 7 [55] and edited in BioEdit v. 7.0.9 [56] when necessary. Phylogenetic analyses were performed using ML, MP and BI as detailed in Dissanayake et al. [57]. The most suitable models for the ML and BI analyses were estimated using MrModeltest v. 2.3 [58] under AIC (Akaike Information Criterion) implemented in PAUP v. 4.0b10. The GTR+I+G model was determined to be the most suitable model for both LSU and ITS gene regions. 

The ML analyses were conducted with RAxML-HPC2 on XSEDE v. 8.2.10 [59] in the CIPRES Science Gateway platform [60] using a GTR+I+G substitution model with 1000 bootstrap replicates. Bayesian inference was performed using MrBayes v. 3.2.6 (GTR+I+G model) [61]. Six simultaneous Markov Chain Monte Carlo analyses were run for 3,000,000 generations. The trees were sampled at every 100th generation. The first 10% of trees were discarded and the remaining 90% were used to calculate the posterior probabilities (PP) in the majority rule consensus tree. PAUP v. 4.0b10 [62] was used to perform the MP analysis for the combined dataset. A heuristic search option with 1000 random replicates and the tree bisection-reconnection (TBR) branch-swapping algorithm was used in the MP analysis. MaxTrees were set to 1000, branches of zero length were collapsed, and all multiple parsimonious trees were saved. Descriptive tree statistics for parsimony—tree length (TL), consistency index (CI), retention index (RI), relative consistency index (RC) and homoplasy index (HI)—were calculated for trees generated under different optimality criteria. Phylograms were visualized with the FigTree v. 1.4.0 program [63] and reorganized in Microsoft PowerPoint (2010). The final alignment and tree were deposited in TreeBASE under the submission ID: 28667 (http://www.treebase.org; accessed on 20 August 2021). 

### 2.2. Molecular Clock Analysis

Divergence times were estimated using BEAST 1.8.4 [64]. The XML input file was prepared using BEAUTI v. 1.8.4. The substitution model, clock model and tree prior were set as linked. The GTR+I+G model was used as the nucleotide substitution model. An uncorrelated relaxed clock model [65] with the log-normal distribution rates was used for the analysis. Yule speciation process birth rate was used for the tree prior starting from a randomly generated tree. The crown age of *Botryosphaeriales* was set as 110 Mya (SD = 5 Mya) [7]. 

BEAST analyses were run for 60 million generations. Log parameters and trees were sampled every 10,000th generation. Tracer v. 1.6 [66] was used to check that effective sample sizes (ESS) were greater than 200. The first 10% of the trees were discarded and the remaining 5,400 trees were used to generate the maximum clade credibility (MCC) tree using LogCombiner v1.8.0 and TreeAnnotator v1.8.0. The resulting trees were viewed with FigTree v.1.4.0 [63] and edited in Microsoft PowerPoint (2010). 

### 2.3. Ancestral State Reconstructions

Bayesian Binary MCMC in RASP 3.2 (Reconstruct Ancestral State in Phylogenies) [67,68] was used for the ancestral state reconstructions for conidial colour (hyaline or pigmented), conidial septation (septate or aseptate) and nutritional modes (saprobes, pathogens or endophytes). The evolution tree was generated in BEAST 1.8.4 [64] using the parameters given under the molecular clock analysis. *Dothideomycetes* crown group was calibrated using the secondary calibration data (normal distribution, mean = 290, SD = 30, providing a 95% credibility interval of 339 Mya) [69]. *Botryosphaeriales* crown group was calibrated using the secondary calibration data (normal distribution, mean = 110, SD = 5, providing a 95% credibility interval of 118 Mya) [7]. 

BEAST analyses were run for 100 million generations. Log parameters and trees were sampled at every 10,000th generation. MCC tree was generated by discarding the first 10% of the trees (1000 trees). The tree file resulting from the evolution analysis was exported to RASP 3.2. Each terminal in the tree was coded according to Table 2. Bayesian Binary MCMC trees were constructed using the following settings: 50,000 generations sampled every 100 generations, 10 chains and 0.1 temperature. State frequencies and among-site rate variation were set as Estimated (F81) and Gamma (+G), respectively. The analysis was applied only to *Botryosphaeriales* species and the character matrix used for this analysis is provided in Table S1. Two hypotheses were assessed as given below:

**Hypothesis 1.**  *All Botryosphaeriales species originated as endophytes and then switched into saprobes when their hosts died or into pathogens when their hosts were under stress.*

**Hypothesis 2.**  *There is a link between the conidial colour and nutritional mode in botryosphaerialean taxa.*

## 3. Results and Discussion

### 3.1. Phylogenetic Analyses

We re-evaluated the phylogenetic relationships within families of *Botryosphaeriales* based on LSU and ITS sequence data. In our preliminary phylogenetic analyses, we used sequence data from LSU, ITS and *tef*1 gene regions. Based on the *tef*1 resolution, *Pseudofusicoccum* and *Phyllosticta* formed separate groups within *Phyllostictaceae*, while *Saccharataceae* did not form a well-separated clade. Therefore, our final phylogenetic analyses were performed based on LSU and ITS sequence data. 

**Figure 3:**
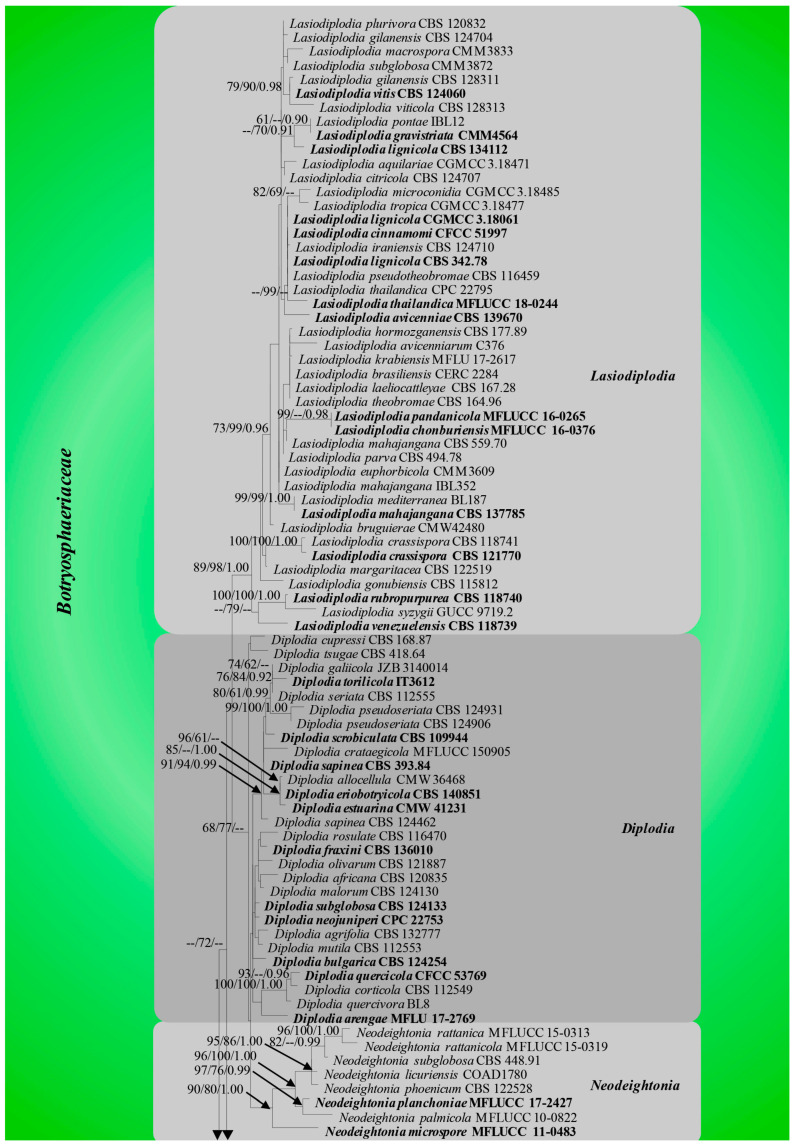

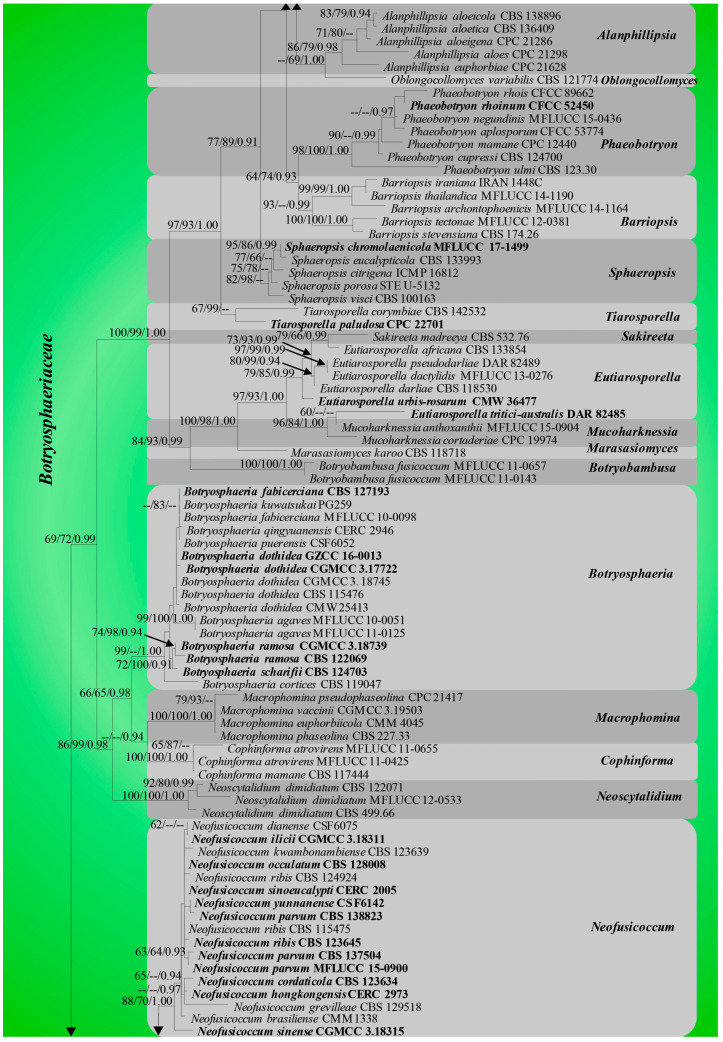

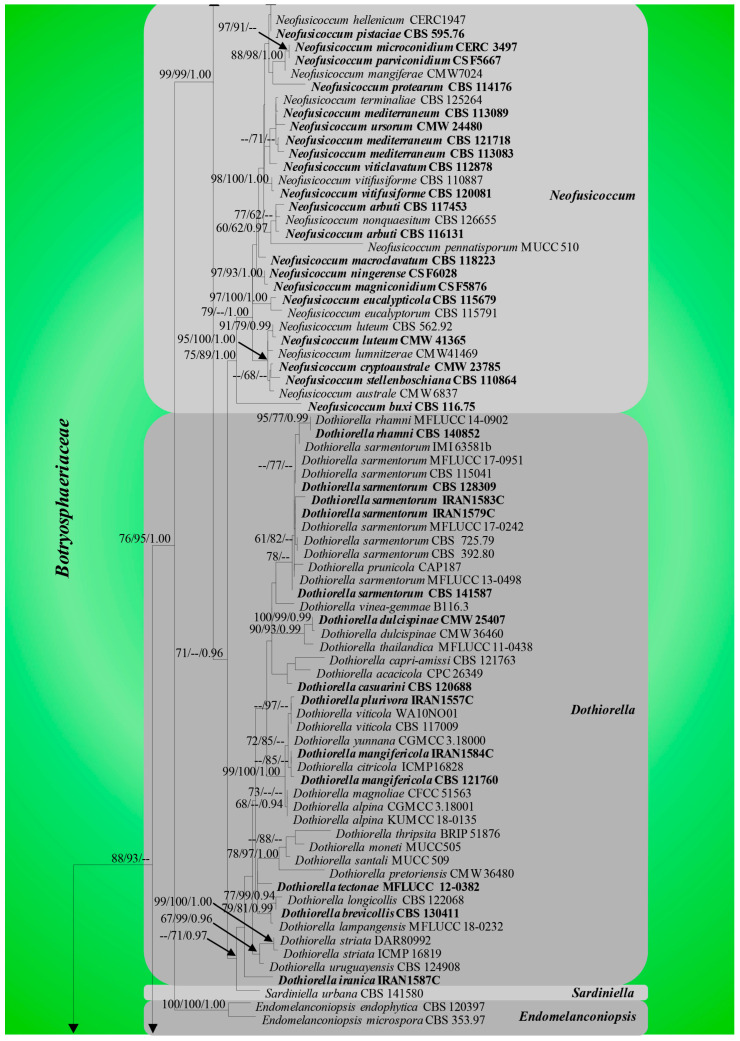

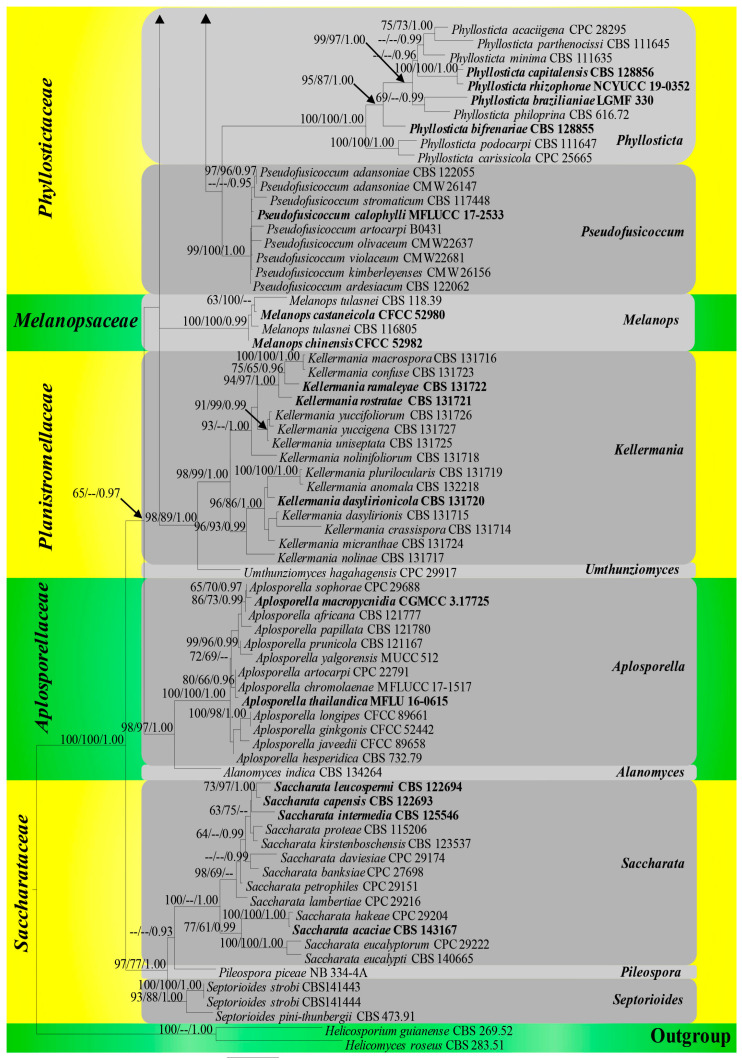
Phylogram generated from ML analysis based on combined LSU and ITS sequence data. ML and MP bootstrap support values ≥ 60% and Bayesian posterior probabilities (PP) ≥ 0.90 are mentioned at the nodes as ML/MP/PP. Strain numbers are noted at the end of the species name. The tree is rooted to *Helicosporium guianense* (CBS 269.52) and *Helicomyces roseus* (CBS 283.51).

The combined dataset consisted of 306 strains, representing botryosphaerialean taxa (*Aplosporellaceae* = 14, *Botryosphaeriaceae* = 236, *Melanopsaceae* = 4, *Phyllostictaceae* = 19, *Planistromellaceae* = 16, *Saccharataceae* = 17) and two outgroup taxa, *Helicosporium guianense* (CBS 269.52) and *Helicomyces roseus* (CBS 283.51) from *Tubeufiaceae*. The aligned dataset comprised 1452 characters including gaps (LSU = 880, ITS = 572). The best scoring RaxML tree with a final likelihood value of −19,919.245301 is shown in Figure 3. The matrix had 745 distinct alignment patterns with 25.42% undetermined characters or gaps. Estimated base frequencies were obtained as follows: A = 0.240022, C = 0.246955, G = 0.283975, T = 0.229048; substitution rates: AC = 1.641167, AG = 3.230258, AT = 1.788585, CG = 1.459944, CT = 7.210110, GT = 1.000000; gamma distribution shape parameter: α = 0.256314. 

In the MP analysis, 775 characters were constant; 173 variable characters were parsimony-uninformative and 740 (37.28 %) characters were parsimony-informative. The most parsimonious tree resulted in the following parameters: TL = 6681, CI = 0.261, RI = 0.825, RC = 0.216, HI = 0.739 (for individual loci, parameters were obtained as follows: LSU, TL = 882, CI = 0.385, RI = 0.866, RC = 0.334, HI = 0.615; and ITS, TL = 2303, CI = 0.283, RI = 0.853, RC = 0.241, HI = 0.717). The average standard deviation of split frequencies was 0.001 after 3,000,000 generations. In the phylogenetic analyses, *Aplosporellaceae*, *Melanopsaceae*, *Planistromellaceae* and *Saccharataceae* segregated with strong bootstrap support values while, *Botryosphaeriaceae* and *Phyllostictaceae* showed moderate bootstrap support (Figure 3). 

Phillips et al. [7] also constructed an ML tree for *Botryosphaeriales* using ITS and LSU sequences. However, except for *Botryosphaeriaceae* and *Phyllostictaceae*, the arrangement of *Aplosporellaceae*, *Melanopsaceae*, *Planistromellaceae* and *Saccharataceae* in the phylogenetic tree is different from this study. Phillips et al. [7] included 100 strains belonging to 28 genera in *Botryosphaeriales* in their analyses, while 306 *Botryosphaeriales* strains in 32 genera were used in our study. Even though we used the same loci as Phillips et al. [7], the sequence alignment was affected by the population size of the samples. This could account for the topological differences in the ML trees of the two studies. 

### 3.2. Divergence Times

**Figure 4:**
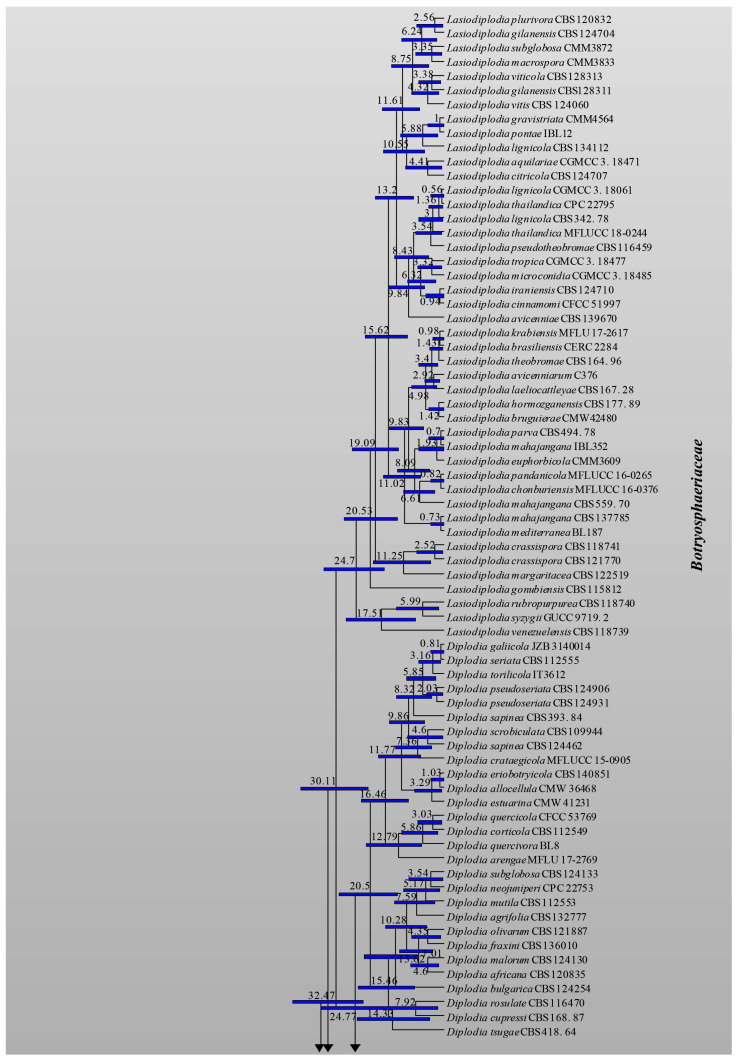

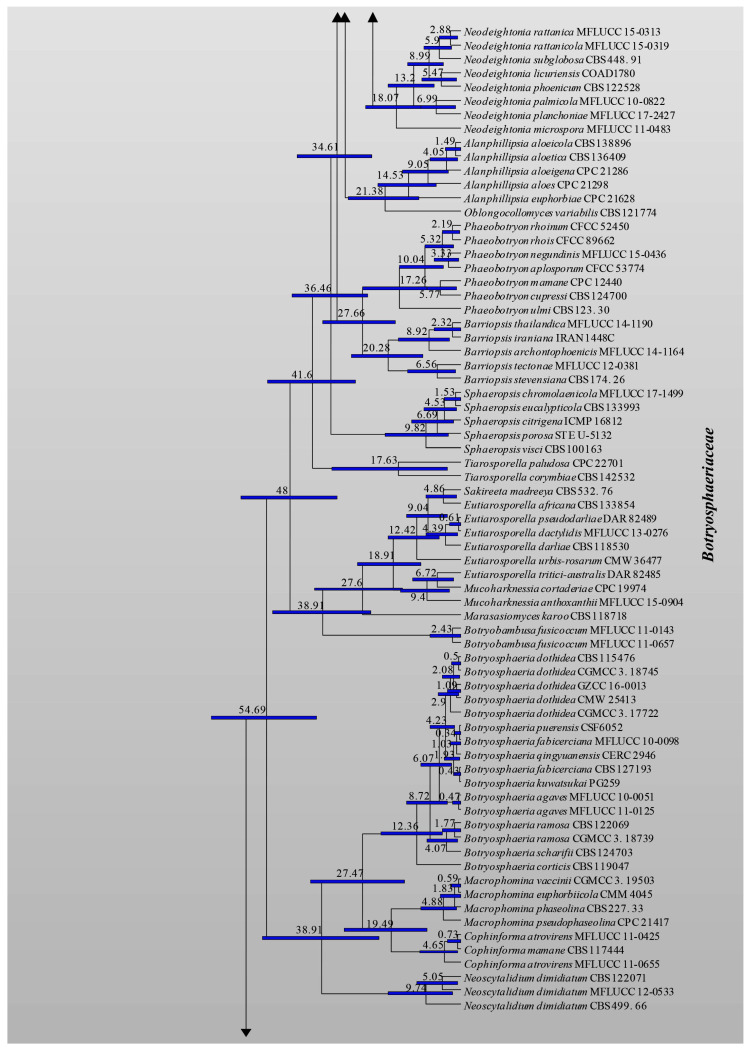

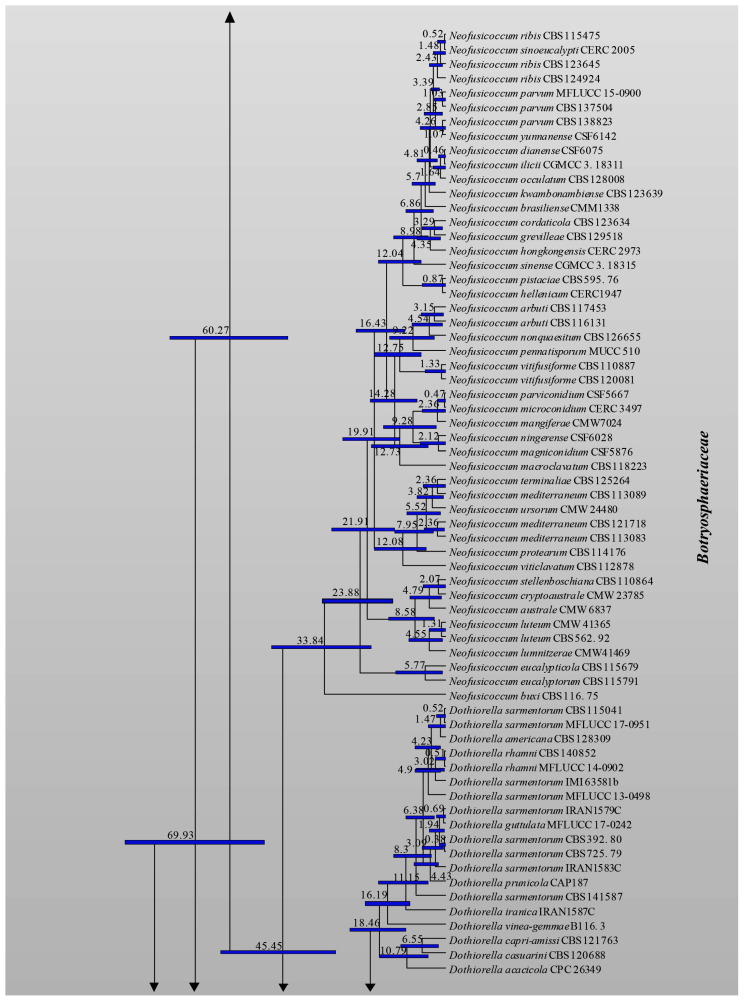

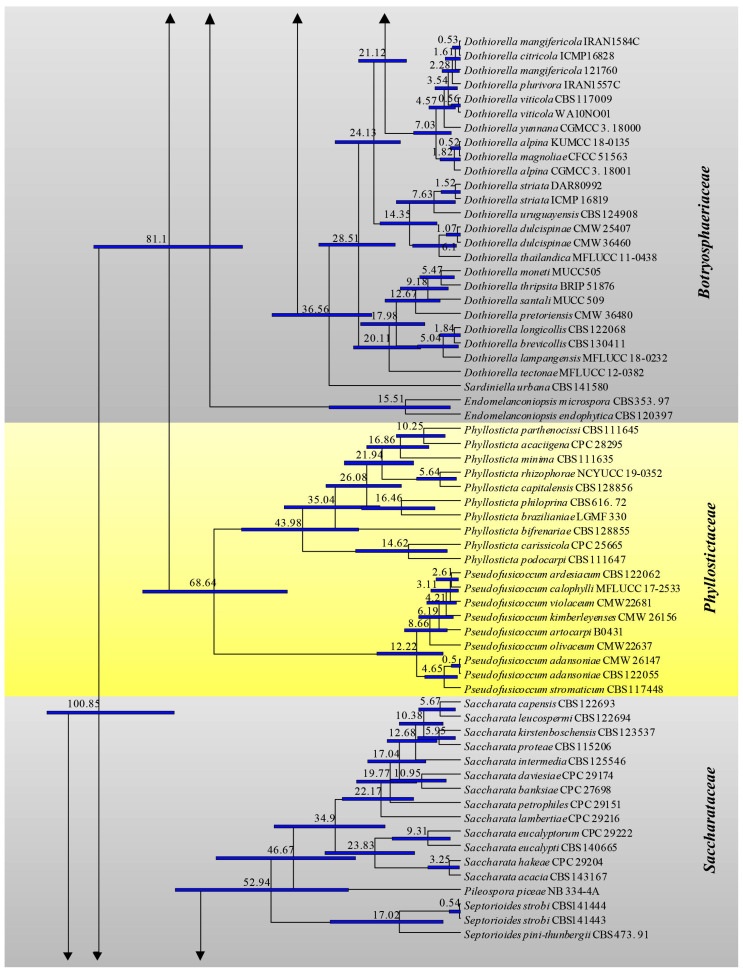

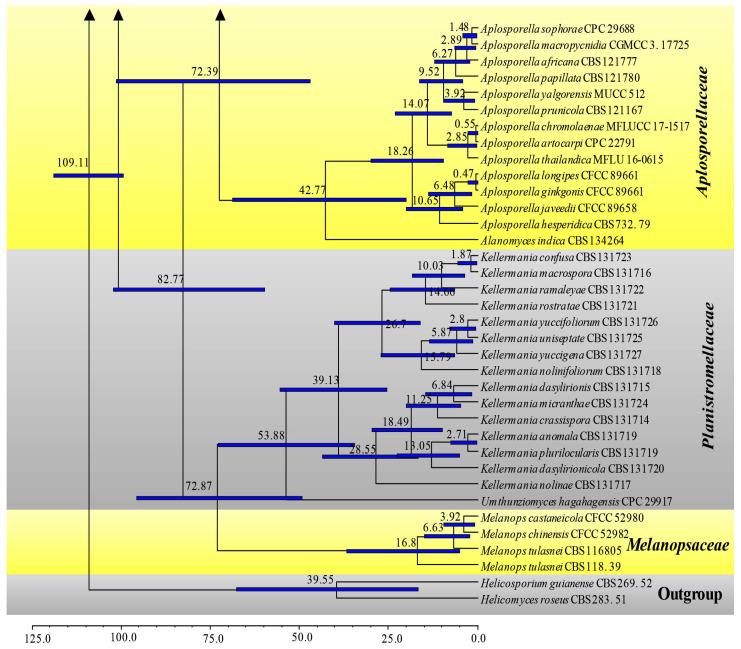
Maximum clade credibility (MCC) tree for LSU and ITS sequence data using a GTR+G+I nucleotide substitution model. The tree was calibrated by setting the crown age of *Botryosphaeriales* at 110 Mya. Values at the nodes are given in millions of years and the blue bars indicate standard deviations.

The topology of the MCC tree (Figure 4) resulting from the evolutionary analysis was similar to the topologies of ML, BI and MP trees. Based on evolutionary analysis, all six families were established during the Cretaceous period. *Botryosphaeriaceae* and *Phyllostictaceae* diversified during the Cretaceous period, while the remaining four families diversified during the Paleogene and Neogene periods in the Cenozoic era (0–66 Mya). The crown and stem ages for each family are tabulated in Table 3. 

Previously, several studies were conducted to perform the divergence time estimations for *Botryosphaeriales* [4,7,70]. The number of taxa, gene regions and calibration points they used and the resulting crown and stem ages are given in Table 4. 

**Figure 5:**
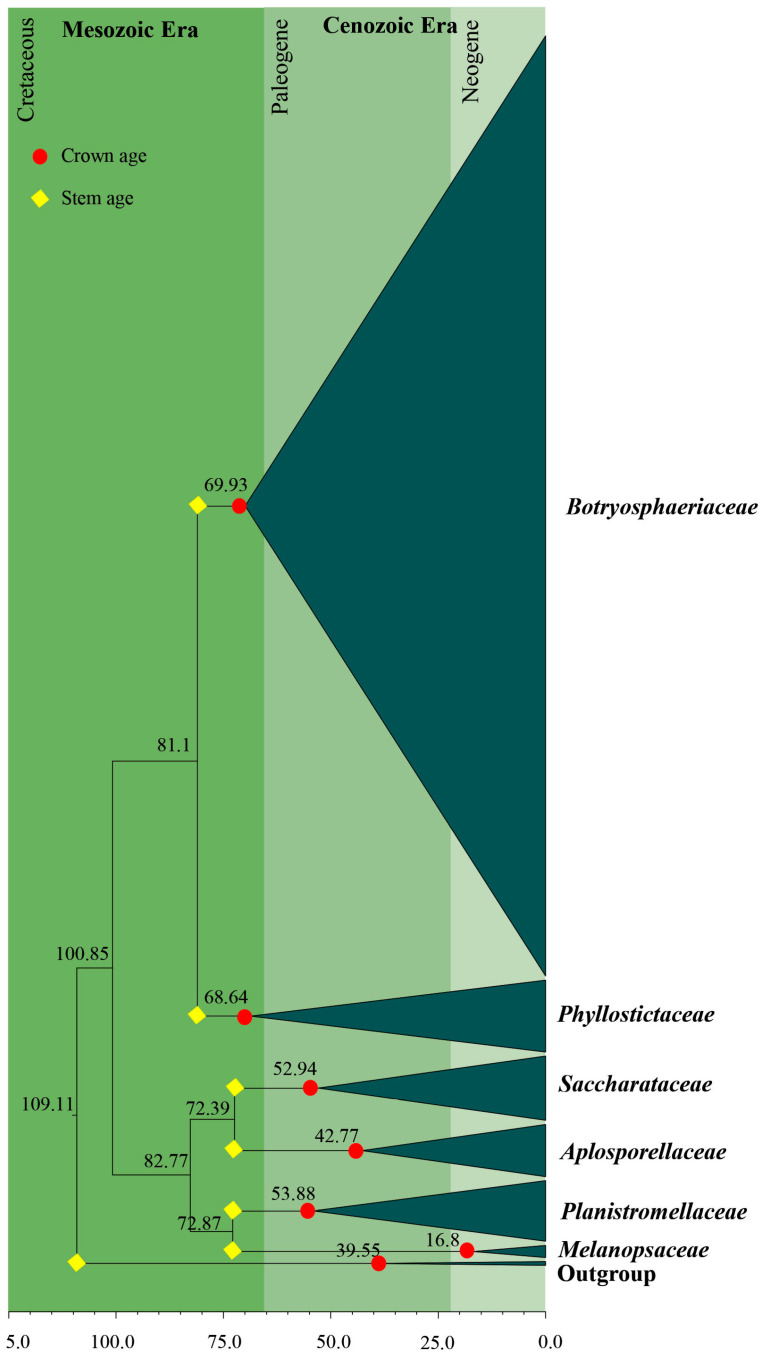
Diagram representing the evolution of families in *Botryosphaeriales* with crown age and stem age.

Previous studies of Slippers et al. [4] and Liu et al. [71] revealed that *Botryosphaeriales* originated 103 (45–188) Mya. Liu et al. [70] reported the crown age of this order as 114 (73–166) Mya, while Phillips et al. [7] considered it to be at 110 Mya. In our analysis, we used 110 Mya to calibrate *Botryosphaeriales*. According to results of our analysis, *Botryosphaeriales* originated at 109 (99–119) Mya (Figure 4). Generally, the diversification of *Botryosphaeriales* may have occurred during the Cretaceous period associated with a rapid diversification of angiosperms (flowering plants). Liu et al. [70] suggested that orders of *Dothideomycetes* evolved within 100–220 Mya (crown age) and according to our study, *Botryosphaeriales* evolved within this range. The evolution of families in *Botryosphaeriales* is illustrated in Figure 5. 

Slippers et al. [4] used SSU, LSU, ITS, *tef*1, *β-tubulin* and mtSSU gene regions for the molecular clock dating analysis of *Botryosphaeriales*, while Liu et al. [70] used LSU, SSU, *tef*1 and *rpb2* for their analysis (Table 4). However, Liu et al. [70] performed their analysis for *Dothideomycetes* and used both secondary data and fossil data for calibrations. In both studies, most of the crown and stem ages are relatively lower than Phillips et al. [7] and this study (Table 4).

In Phillips et al. [7] and this study, the same gene regions and same calibration points were used to perform the divergence time estimation for *Botryosphaeriales* with a different number of taxa. Similar results are shown for crown age and stem age in both studies. However, there were slight differences (Table 4). Therefore, further studies are required to investigate how the number of taxa effect crown and stem ages in divergence time estimation. 

Previously, *Pseudofusicoccum* was placed in *Botryosphaeriaceae* [4]. Subsequently, Yang et al. [6] showed that *Pseudofusicoccum* forms a separate clade at the base of the family *Botryosphaeriaceae* and suggested it as a separate family in *Botryosphaeriales*. Phillips et al. [7] accepted *Pseudofusicoccum* in *Phyllostictaceae* with support from the morphology of asexual morphs. The ML and MCC trees (Figure 3 and Figure 4) obtained in this study also show that *Pseudofusicoccum* group into *Phyllostictaceae* as in Phillips et al. [7]. Therefore, this study accepts *Pseudofusicoccum* as one of the genera in *Phyllostictaceae*. Liu et al. [70] suggested that families should have evolved between 20–100 Mya (crown age) in general. According to our study, all six families in *Botryosphaeriales* have evolved within this time frame (Table 3). Thus, our results support the establishment of the order *Botryosphaeriales* and accept *Aplosporellaceae*, *Botryosphaeriaceae*, *Melanopsaceae*, *Phyllostictaceae*, *Planistromellaceae* and *Saccharataceae* as families in this order.

### 3.3. Ancestral State Reconstructions

**Figure 6:**
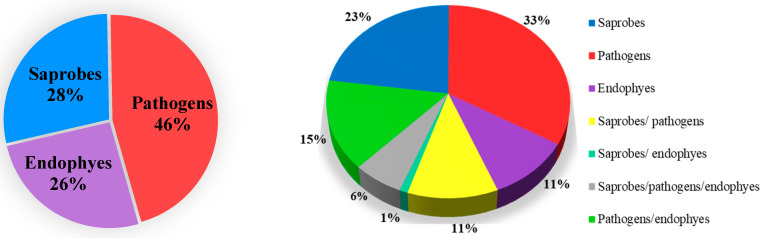
Nutritional modes recorded in *Botryosphaeriales* taxa.

In ancestral state reconstructions, morphological or ecological data are mapped on molecular phylogenetic information generated from ML, MP and BI approaches [51]. Ancestral state reconstructions for conidial colour and septation, and nutritional mode evolution in botryosphaerialean taxa used the evolution tree results from BEAST 1.8.4 [64] under the Bayesian Binary MCMC method in RASP software (Figure 6) [67,68]. Three different nutritional modes were considered, namely endophytic, pathogenic and saprobic to assess the evolution of nutritional mode analysis. Some botryosphaerialean taxa are hemibiotrophic (*Botryosphaeria dothidea*), while some are necrotrophic (*Phaeobotryon negundinis*). Therefore, we included hemibiotrophic and necrotrophic modes under the pathogenic mode. Two hypotheses were tested in the current study as given in the methodology. 

#### 3.3.1. Ancestral State Reconstructions on Nutritional Modes of *Botryosphaeriales* Taxa

This analysis was conducted to assess the hypothesis that “All *Botryosphaeriales* species originated as endophytes and then switched into saprobes when their hosts died or into pathogens when their hosts were under stress”. This analysis is based mainly on the results of previous studies (Table S1). The endophytic nutritional mode in *Ascomycota* originated around 590–467 Mya in the stem lineage of *Pezizomycotina*, and many lineages show an endophytic ancestral character [72,73]. Based on our analysis, *Dothideomycetes* evolved with an endophytic ancestral nutritional mode around 250 Mya. They switched from endophytic to saprobic around 230 Mya. The supercontinent drift began in the Paleozoic (541–251 Mya), followed by the disintegration of the Pangea plate in the Middle Jurassic (176–161 Mya) [74]. These events resulted in the formation of continental amalgamation in the early Cretaceous, and plants were widely spread during this period. The interaction between plants and fungi facilitates the fungal colonization on land plants and their ability to adapt to different environmental conditions [75,76]. This may influence the *Dothideomycetes* to switch their nutritional mode from endophytes to saprobic during this period. Promputtha et al. [77] stated that many endophytes have the capacity to degrade cellulose and lignin. Therefore, they became part of the decomposer community by switching into saprobes, increasing saprobic diversity and decomposition rates [78].

This study revealed that most of the botryosphaerialean taxa were pathogens (46%) and few were recorded as endophytes (26%) (Figure 6). Among the 306 botryosphaerialean taxa included in the current study, 94 taxa were recorded exclusively as pathogens (31%), while 68 and 32 taxa were recorded exclusively as saprobes (22%) and endophytes (10%), respectively (Figure 6). Results of this study indicate that a pathogenic/saprobic ancestral nutritional mode for *Botryosphaeriales* evolved at around 109 Mya, which was derived from a pathogenic/endophytic ancestor around 136 Mya. Later, this pathogenic/saprobic ancestral nutritional mode diversified into endophytic/pathogenic/saprobic at 100 Mya at the late epoch of the Cretaceous period. The results of this analysis could not provide support for our hypothesis, which indicates the endophytic mode to be the ancestral nutritional mode. These results can be influenced by the fact that most of the botryosphaerialean taxa recorded and used in this study are pathogens and saprobes (Table S1), which will be discussed further.

Endophytic species were recorded from all the families of *Botryosphaeriales,* but the number of studies is very low compared to the saprobic and pathogenic (Table S1). The unbalanced taxon sampling for the analysis may exhibit a bias towards the pathogenic and saprobic modes. Another reason may be that for most of the species in this order, it is very common to be isolated as a pathogen or a saprobe. This is because pathogenic is the form where they become obvious, and researchers have focused their efforts on studies at this stage for economic reasons. Therefore, we do not have evidence to identify their initial nutritional mode and whether they experience nutritional mode shifts during their life cycle. For example, *Botryosphaeria dothidea* has been commonly reported as a serious plant pathogen, and has also been isolated as an endophyte [10]. Therefore, studies are needed to check whether we can isolate a species as a pathogen and also as an endophyte from the same host at different times. Similar to the current study, where unbalanced taxon sampling exhibits a bias towards the pathogenic and saprobic modes, a study conducted on *Pucciniomycotina* has shown mycoparasitism as the ancestral nutritional mode, while the mycoparasitic mode seems to be the most widespread in *Pucciniomycotina* [79,80,81]. This demonstrates that the taxon sampling for the study and the family composition might influence the results of ancestral nutritional mode studies. 

In addition, warm environmental conditions that existed in the early epoch of the Cretaceous period (145–100.5 Mya) might also influence the ancestral endophytic taxa to become saprobic. Therefore, this event of diversifying endophytic taxa to saprobic should have occurred at around 109 Mya. In a fossil study at the Deccan Intertrappean Beds of India, both saprobic and pathogenic fungi were recorded in the late Cretaceous (100.5–66 Ma) [82], providing evidence for the occurrence of saprobic and pathogenic fungi in the late Cretaceous period other than endophytes. These may be the reasons why *Botryosphaeriales* have a pathogenic/saprobic nutritional mode in their ancestors. However, it is difficult to identify the fungal endophytes in fossil materials because it is hard to determine whether the host was alive and functioning or was going through senescence or decay at the time of colonization [83,84]. 

A study conducted by Schoch et al. [85] using the phylogeny of extant lineages found saprobic and parasitic modes among the ancestral characters of *Pezizomycotina*. Similarly, Savile [86] proposed the existence of parasitic fungi on vascular plants in the early stages of territorialization. Using this evidence, Lücking et al. [87] formulated the ‘green scenario’ which stated that parasitic fungi from freshwater bodies co-evolved with the ancestors of land plants and diversified to many lifestyles [81]. Together, with all these facts and evidence for the existence of pathogenic and saprobic ancestral modes, we can explain that the pathogenic/saprobic nutritional mode resulted as the ancestral nutritional mode for *Botryosphaeriales* in our analysis.

**Figure 7:**
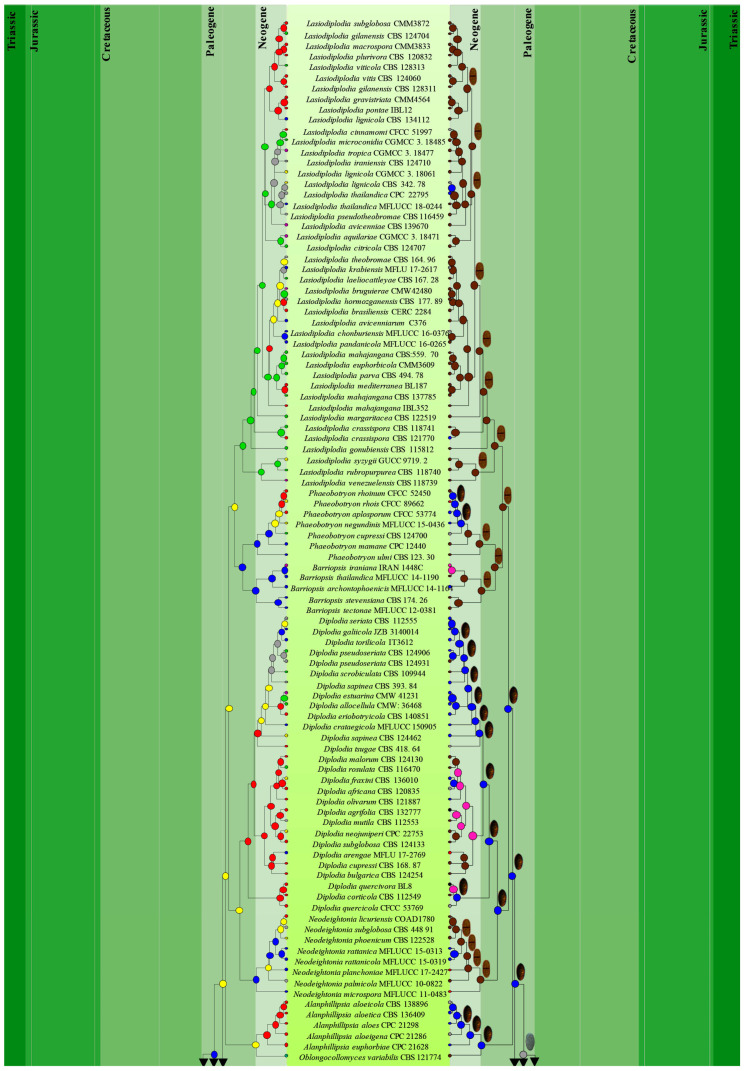

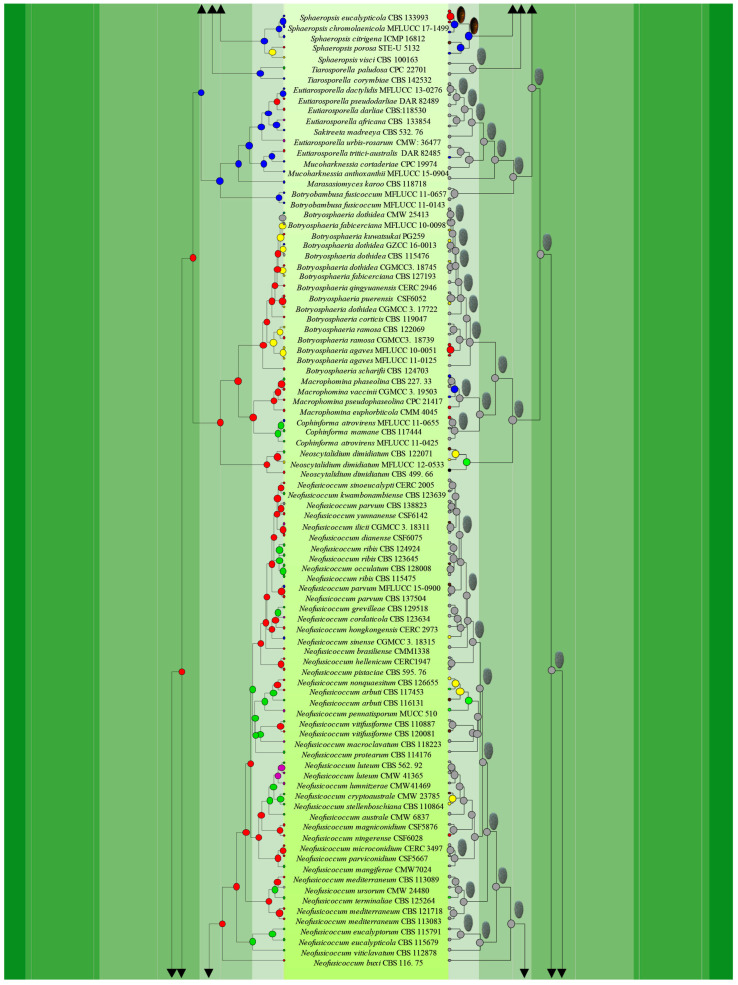

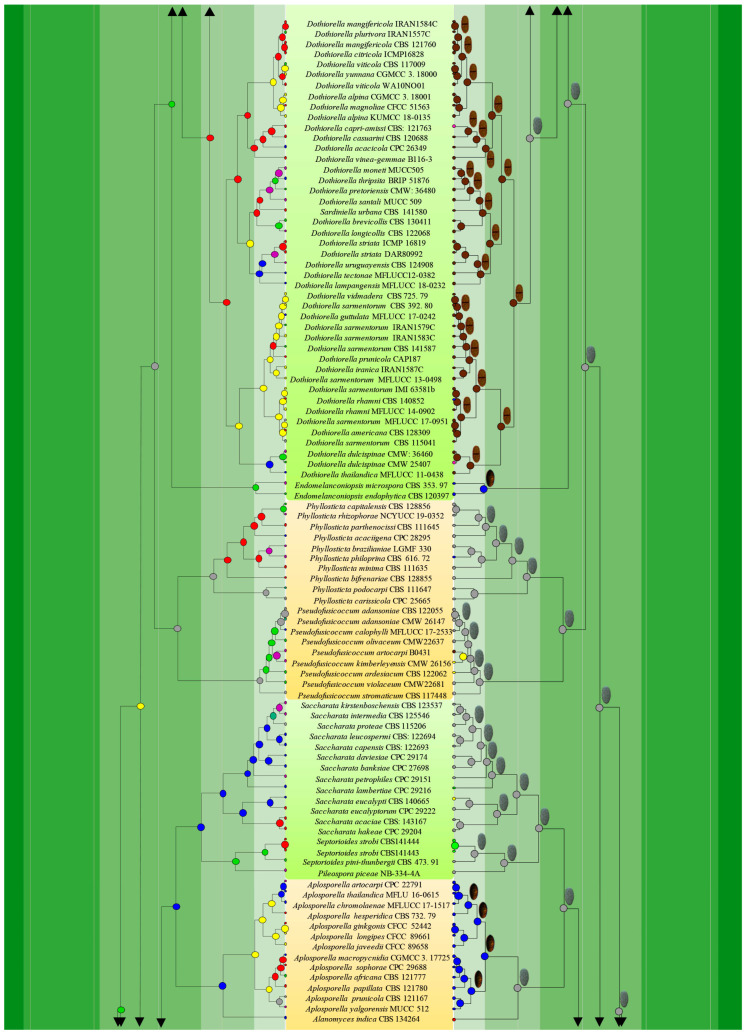

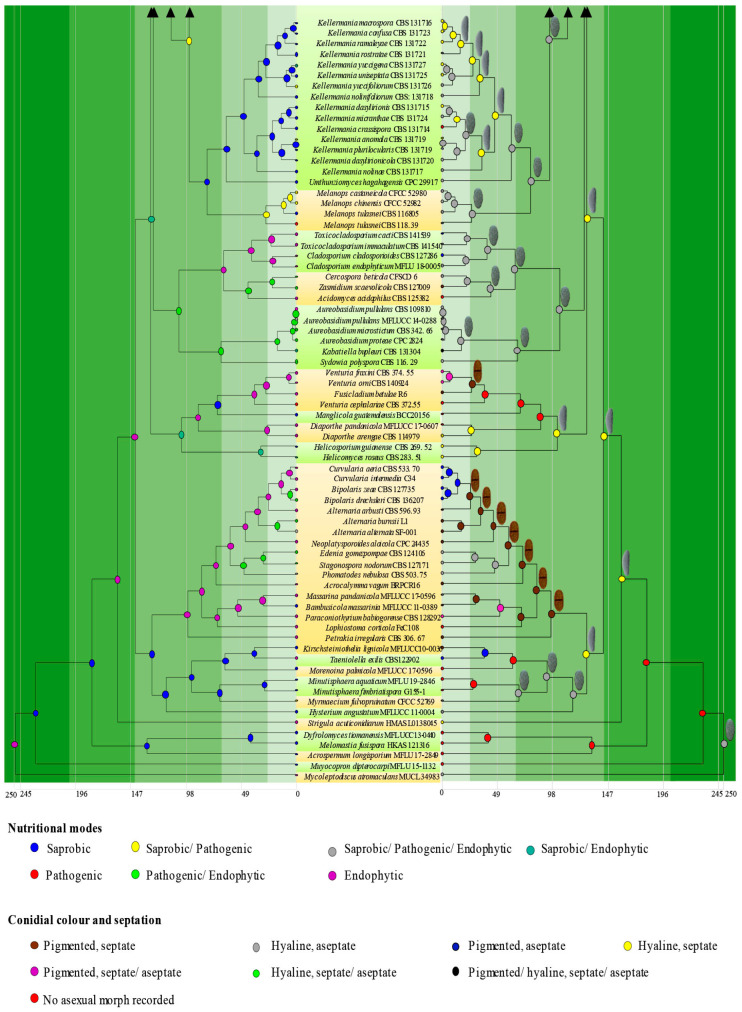
Ancestral character state analysis for nutritional mode (left) and conidial colour and septation (right) in *Botryosphaeriales*, using Bayesian Binary MCMC approaches. Pie charts at terminals show the most likely states (MLS) only and the internal nodes represent the marginal probabilities for each ancestral area.

As time progresses, the nutritional mode of botryosphaerialean taxa diversified into all three nutritional modes, i.e., endophytes/saprobes/pathogens (around 100 Mya), suggesting multiple switching events during their evolution (Figure 7). Flowering plants and other flora, such as deciduous trees (modern plants), ferns and grasses were abundant during the Cretaceous and Paleogene periods [88,89]. Angiosperms diversified rapidly during the Cretaceous period and *Botryosphaeriaceae* species are mostly diverse on Angiosperms [4]. Batista et al. [90] mentioned that the high host diversity may affect the fungal diversity in different plant functional groups. Therefore, the diversity of the plant hosts is one of the reasons for the change in the nutritional modes in botryosphaerialean species. This is also evident in other fungal lineages. Some studies have shown that the nutritional mode switch from the ancestral insect-parasitic or plant-pathogenic fungi to endophytic ascomycetes [91,92], and some show a switch from lichen-forming, endolichenic and saprotrophic fungi to endophytic fungi [93].

Stress or pressure on plants is a factor that changes with climatic conditions or extreme weather conditions (high temperatures, cold, drought or extreme rain) and the effect of herbivores or pests on plants [10]. Under this stress or pressure, many endophytic fungi became pathogenic to plants [10,73]. According to the results of our analysis, endophytic/pathogenic botryosphaerialean taxa diversified into pathogenic taxa at around 78 Mya in the late epoch of the Cretaceous (Figure 7). During the Cretaceous period, warmer and humid environments that existed caused increased stress on plants and led to botryosphaerialean taxa changing their nutritional mode from endophytic to pathogenic. Endophytic taxa also become saprobic when environmental conditions are unfavourable to the host or when the host dies [73,94]. 

In their study, Hyde et al. [73] suggested two scenarios for the evolution of *Diaporthomycetidae*, i.e., (1) The ancestors of *Diaporthomycetidae* had endophytic lifestyles that colonized inside the plants similar to some aquatic hyphomycetes that also share endophytic ancestors. These endophytic fungi become active when the plants are under stress or senesced and convert into either saprobes to decay the dead plant parts or pathogens to cause disease. (2) The ancestors of *Diaporthomycetidae* had non-specific saprobic lifestyles and at some point, they became plant pathogens in specific plants to cause diseases. Based on our results, we also can accept the first hypothesis for the class *Dothideomycetes*. However, for *Botryosphaeriales*, none of the hypotheses are applicable. 

However, with only a few studies on endophytic botryosphaerialean taxa, we were unable to provide conclusive evidence for our hypothesis that the endophytic nutritional mode could be ancestral for *Botryosphaeriales* species, and later they diversified into saprobic and pathogenic modes. Therefore, further studies are required related to endophytic species in *Botryosphaeriales* to investigate this hypothesis.

#### 3.3.2. Ancestral State Reconstructions for Conidial Colour and Septation in *Botryosphaeriales* Taxa

In this analysis, we assessed our second hypothesis that “There is a link between the conidial colour and nutritional mode in botryosphaerialean taxa”. The evolution of two morphological characters, conidial colour and septation, was reconstructed by employing the tree generated from the evolution analysis (Figure 7). Two parameters were considered: hyaline and pigmented. All light brown, brown and dark brown conidia were considered as pigmented. Septate and aseptate parameters were used for conidial septation. All conidia with one or more septa were included under the septate parameter. 

Ancestral character analyses of conidial colour and septation indicate the hyaline and aseptate conidia as the common ancestral character in both *Botryosphaeriales* and *Dothideomycetes*. This is not the first study to show hyaline fungal structures as an ancestral form. The hyaline appressoria are considered ancestral in appressorial fungi [54]; similarly, hyaline ascospores in *Xylariomycetidae* are regarded as ancestral [95]. 

At around 49 Mya and 47 Mya in the Eocene of the Paleogene (33.9–56 Mya), botryosphaerialean taxa diversified their conidial colour from hyaline to pigmented and conidial septation from aseptate to septate, respectively. Hyaline to pigmented and septate to aseptate conidia occur among taxa in *Botryosphaeriaceae*. Most of the conidia in *Phyllostictaceae* are hyaline and aseptate, while few are pigmented and septate [3,39,41,96]. *Phyllosticta philoprina* and *Pseudofusicoccum artocarpi* have pigmented conidia and among them, *P*. *artocarpi* has septate conidia (Table S1). Both *Pseudofusicoccum ardesiacum* and *P*. *kimberleyensis* have hyaline, septate conidia [19,97]. Hyaline and pigmented conidia occur in *Melanopsaceae* and *Aplosporellaceae*, respectively, and are aseptate in both families. *Planistromellaceae* have both septate and aseptate hyaline conidia. In *Saccharataceae*, most of the species have hyaline, aseptate conidia, while few have hyaline, septate conidia (Table S1). 

The fossil records of *Diplodia* (*Sphaeropsis*) have been recorded from permineralized chert from the Deccan Intertrappean bed, India [98]. Two-celled spores 13 μm long and thick-walled, oval and pycnidia have been recorded in the permineralized specimens of *D*. *intertrappea* [99]. Therefore, septate conidia were recorded in *D*. *intertrappea* in ancient times. Similarly, fossil records of *Diplodites rodei* (Basionym: *Diplodia rodei*) and *D*. *sahnii* (Basionym: *Diplodia sahnii*) have been recorded from the Mohgaonkalan locality in Chhindwara District, Madhya Pradesh, India [100]. These fossils belonged to the Late Cretaceous period [100]. According to the fossil records, *Diplodites* had one-septate dark brown or aseptate light brown conidia [100,101]. Fossils of *Diplodites* are morphologically similar to the extant fungi of *Diplodia*, *Dothiorella* and *Macrophoma* [100]. This provides evidence that in the late Cretaceous period, ancestors of *Diplodia* had aseptate or one-septate, light to dark brown conidia [100].

The evolutionary study (Figure 4) indicates *Botryosphaeriales* originated and evolved during the Cretaceous (66–145 Mya) and Paleogene periods (23–66 Mya). A warm environment existed during the Cretaceous period, which may be in response to volcanic activity and increased atmospheric greenhouse gas concentrations [88]. The temperature of the sea surface during the Cretaceous period varied between 37–42 °C [88]. During the Paleogene period, the temperature dropped to about 23–29 °C (±4.7 °C) and formed a cool and dry environment [89]. According to Hagiwara et al. [102], temperature mainly affects conidial pigmentation. As they suggested, most species produce pigmented conidia at 25 °C or 37 °C [102]. 

At around 49 Mya, some members of *Botryosphaeriales* diversified from hyaline conidia to pigmented, while others remained as hyaline. Melanized spores survive under extreme environmental conditions, such as excessive heat or cold, extremely dry conditions, extreme pH or osmotic conditions, hypersaline environments, polychromatic radiation, radionuclides and UV radiation [47]. Non-pigmented spores die under hard UV radiation within a few minutes, but melanized spores survive [46,47]. Based on these observations and our results, we conclude that hyaline conidia diversified to pigmented during the Eocene epoch of the Paleogene period for survival under harsh environmental conditions, such as high temperature variation. The Paleocene–Eocene was considered as the most significant time period of global warming and was followed by a long cool and dry period [103].

In addition, we evaluated sexual morph characters, i.e., ascospore colour and septation in preliminary studies. Due to a lack of variation and representative data, reliable results were not obtained. Therefore, we did not include ascospore characters for the ancestral character analyses in this study.

**Figure 8:**
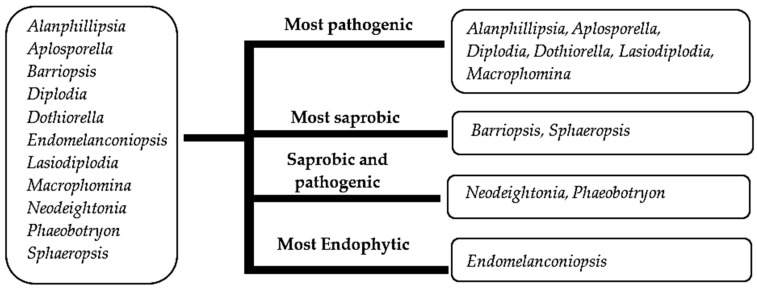
Genera with pigmented conidia and their nutritional modes.

Belozerskaya et al. [47] showed that both saprobic and pathogenic taxa have melanized conidial walls that appeared as pigmented conidia. Our study confirmed that there was link between conidial colour and nutritional mode in botryosphaerialean taxa, which supports our second hypothesis. Based on the results of the ancestral character analyses, the genera with pigmented conidia show pathogenic and saprobic nutritional modes (Figure 8). Exceptions to this are *Endomelanconiopsis* species that have pigmented conidia, even though most of them are endophytic (Figure 8). Most *Botryosphaeria* and *Neofusicoccum* species are pathogenic and unlike others, most of them have hyaline conidia. Therefore, our second hypothesis is not applicable for all the genera in *Botryosphaeriales*. Most of the pathogenic genera have pigmented conidia in this order.

## 4. Conclusions

In this study, updated phylogenetic analyses (ML, MP and BI), evolutionary divergence times, ancestral character analyses for conidial colour and septation and nutritional mode analyses are provided for all families in *Botryosphaeriales*. Based on our findings, we conclude: (1) Six families, namely, *Aplosporellaceae*, *Botryosphaeriaceae*, *Melanopsaceae*, *Phyllostictaceae*, *Planistromellaceae* and *Saccharataceae* in this order were well-separated in our phylogenetic analyses. (2) According to divergence times, *Botryosphaeriales* may have originated in the Cretaceous period in the Mesozoic era, and all six families evolved during this period. Later, *Botryosphaeriaceae* and *Phyllostictaceae* divided into genera during the Mesozoic era (66–251.90 Mya), while other families divided during the Cenozoic era (66 Mya–present). Thus, the results of our divergence times estimation also support establishing *Botryosphaeriales* as an order and accepting *Aplosporellaceae*, *Botryosphaeriaceae*, *Melanopsaceae*, *Phyllostictaceae*, *Planistromellaceae* and *Saccharataceae* as families in this order. (3) Ancestral character analyses of conidial colour and septation and nutritional mode revealed that the common ancestor in *Botryosphaeriales* had hyaline, aseptate conidia and a pathogenic/ saprobic nutritional mode. Later, at 100 Mya in the late Cretaceous period, this pathogenic/saprobic ancestral nutritional mode diversified into an endophytic/pathogenic/saprobic. Botryosphaerialean taxa diversified their conidial colour from hyaline to pigmented and conidial septation from aseptate to septate in the Paleogene period. During evolution, *Botryosphaeriales* species diversified their conidial colour, septation and nutritional mode in response to harsh environmental conditions. Here, we investigated the hypothesis that the common ancestor of botryosphaerialean taxa had an endophytic nutritional mode and later deviated into saprobic or pathogenic when their hosts died or were under stress. However, due to the very low number of endophytic studies compared to the saprobic and pathogenic data, it was not possible to draw strong conclusions for the above hypothesis. This study revealed that further studies of endophytic taxa are required and suggested that the taxon sampling and the family composition might have affected the results of ancestral nutritional mode studies. (4) We also tested another hypothesis that related to the link between conidial colour and nutritional mode in botryosphaerialean taxa. Under this hypothesis, we considered the linkage between conidial pigmentation and the pathogenicity of *Botryosphaeriales* taxa. We suggest that the above correlation is applicable for most of the pathogenic genera in *Botryosphaeriales*, but not for all genera.

## Figures and Tables

**Table 1:** Ascospore and conidial morphology (colour and septation) in *Botryosphaeriales* families.

Character			*Aplosporellaceae*	*Botryosphaeriaceae*	*Melanopsaceae*	*Phyllostictaceae*	*Planistromellaceae*	*Saccharataceae*	References
Colour	Ascospore	Pigmented							[3,4,7,36,49]
		Hyaline							
	Conidia	Pigmented							[3,4,50]
		Hyaline							
Septation	Ascospore	Septate							[7]
		Aseptate							
	Conidia	Septate							[7,11,19,50]
		Aseptate							

**Table 2:** Parameters of each character used in ancestral state reconstructions.

Character	Parameter
Conidial colour	Hyaline (A), pigmented (B) and no asexual morph recorded (C)
Conidial septation	Aseptate (A), septate (B) and no asexual morph recorded (C)
Nutritional mode	Saprobes (A), pathogens (B) and endophytes (C)

**Table 3:** Divergence times of crown age and stem age of families of *Botryosphaeriales*.

Family	Divergence Times of Crown Age (Mya)	Divergence Times of Stem Age (Mya)
*Aplosporellaceae*	42.8 (20.1–68.9)	72.4 (46.9–101.2)
*Botryosphaeriaceae*	69.9 (50.5–89.5)	81.1 (60.9–102.1)
*Melanopsaceae*	16.8 (5.1–36.8)	72.9 (49.3–95.7)
*Phyllostictaceae*	68. 6 (48.4–88.4)	81.1 (60.9–102.1)
*Planistromellaceae*	53.9 (34.5–72.7)	72.9 (49.3–95.7)
*Saccharataceae*	52.9 (31.3–79.5)	72.4 (46.9–101.2)

**Table 4:** Details of the divergence times of crown age and stem age of *Botryosphaeriales* families in different studies.

Study		Slippers et al. [4]	Liu et al. [70]	Phillips et al. [7]	This Study
No. of taxa		140	364	100	306
Gene regions		SSU, LSU, ITS, *tef*1, *β-tubulin* and mtSSU (mitochondrial ribosomal small subunit)	LSU, SSU, *tef*1 and *rpb2*	ITS and LSU	ITS and LSU
Calibration/s		Mean = 0.000113 (SD = 0.000006)	Mean = 582.5 Mya (SD = 50.15 Mya) Fossil data 100 Mya (SD = 150 Mya) fossil *Metacapnodiaceae*	Mean = 110 Mya (SD = 5 Mya)	Mean = 110 Mya (SD = 5 Mya)
Divergence time of crown age (Mya)	*Aplosporellaceae*	-	-	40	43
	*Botryosphaeriaceae*	44	44	61	70
	*Melanopsaceae*	-	-	Not estimated	17
	*Phyllostictaceae*	26	27	63	69
	*Planistromellaceae*	38	25	52	54
	*Saccharataceae*	-	28	50	53
Divergence time of stem age (Mya)	*Aplosporellaceae*	57	-	94	72
	*Botryosphaeriaceae*	87	52	94	81
	*Melanopsaceae*	75	-	74	73
	*Phyllostictaceae*	87	50	81	81
	*Planistromellaceae*	75	85	81	73
	*Saccharataceae*	-	114	74	72

## Data Availability

Not applicable.

## Supplementary Materials

The following supporting information can be downloaded at: https://www.mdpi.com/article/10.3390/jof9020184/s1, [104,105,106,107,108,109,110,111,112,113,114,115,116,117,118,119,120,121,122,123,124,125,126,127,128,129,130,131,132,133,134,135,136,137,138,139,140,141,142,143,144,145,146,147,148,149,150,151,152,153,154,155,156,157,158,159,160,161,162,163,164,165,166,167,168,169,170,171,172,173,174,175,176,177,178,179,180,181,182,183,184,185,186,187,188,189,190,191,192,193,194,195,196,197,198,199,200,201,202,203,204,205,206,207,208,209,210,211,212,213,214,215,216,217,218,219,220,221,222,223,224,225,226,227,228,229,230,231,232,233,234,235,236,237,238,239,240,241,242,243,244,245,246,247,248,249,250,251,252,253,254,255,256,257,258,259,260,261,262,263,264,265,266,267,268,269,270,271,272,273,274,275,276,277,278,279,280,281,282,283,284,285,286,287,288,289,290,291,292,293]. Table S1: Nutritional mode variation and conidial characters (colour and septation) and GenBank accession numbers of the sequences used in phylogenetic analysis.
