# Supplementary material for: Re-Evaluating Botryosphaeriales: Ancestral State Reconstructions of Selected Characters and Evolution of Nutritional Modes
Source: J Fungi (Basel). 2023 Jan 29;9(2):184. doi: 10.3390/jof9020184 (PMC9961722; doi:10.3390/jof9020184)
Supplement: Supplementary file 1 [file jof-09-00184-s001.zip › jof-2144279-supplementary.pdf]

Supplementary Table S1. Nutritional mode variation and conidial characters (colour and septation) and GenBank accession numbers of the sequences used in phylogenetic analysis. Type strains are indicated in *.

Species	Strain no.	Nutritional mode	Conidia character		GenBank accession numbers		References
			Colour	Septation	ITS	LSU	
Aplosporellaceae							
Alanomyces indica	CBS 134264	Saprobes	Not observed		HF563622	HF563623	[37]
Aplosporella africana	CBS 121777	Endophytes/ pathogens	Pigmented	Aseptate	EU101315	EU101380	[104,105]
A. artocarpi	CPC 22791	Endophytes	Pigmented	Aseptate	NR_154688	N/A	[106]
A. chromolaenae	MFLUCC 17-1517	Saprobes	Pigmented	Aseptate	MT214340	NG_070506	[107]
A. ginkgonis	CFCC 52442	Pathogens	Pigmented	Aseptate	KM030583	KM030590	[108]
A. hesperidica	CBS 732.79	Pathogens	Pigmented	Aseptate	KX464083	KX464239	[109]
A. javeedii	CFCC 89658	Pathogens/ saprobes	Pigmented	Aseptate	KM030580	KM030587	[104,105]
A. longipes	CFCC 89661	Saprobes/ pathogens	Pigmented	Aseptate	KM030583	KM030590	[110]
A. macropycnidia	CGMCC 3.17725*	Saprobes	Pigmented	Aseptate	NR_154707	N/A	[104]
A. papillata	CBS 121780	Pathogens	Pigmented	Aseptate	KF766197	EU101383	[111]
A. prunicola	CBS 121167	Pathogens/ saprobes/ endophytes	Pigmented	Aseptate	KF766147	KF766315	[104,112,113]
A. sophorae	CPC 29688	Pathogens	Pigmented	Aseptate	KY173388	KY173482	[110]
A. thailandica	MFLU 16-0615*	Saprobes	Pigmented	Aseptate	NR_154722	N/A	[36]
A. yalgorensis	MUCC 512	Endophytes	Pigmented	Aseptate	EF591927	EF591944	[114]
Botryosphaeriaceae							
Alanphillipsia aloeicola	CBS 138896	Pathogens	Hyaline	Aseptate	KP004444	KP004472	[115]
A. aloeigena	CPC 21286	Pathogens	Hyaline	Aseptate	KF777137	KF777193	[110]
A. aloes	CPC 21298	Pathogens	Pigmented	Aseptate	KF777138	KF777194	[110]
A. aloetica	CBS 136409	Pathogens	Pigmented	Aseptate	KF777139	KF777195	[110]
A. euphorbiae	CPC 21628	Pathogens	Pigmented	Aseptate	KF777140	KF777196	[110]
Barriopsis archontophoenicis	MFLUCC 14-1164	Saprobes	Pigmented	Aseptate	KX235306	KX235307	[116]
B. iraniana	IRAN 1448C	Endophytes	Pigmented	Aseptate/ septate	FJ919663	KF766318	[117]
B. stevensiana	CBS 174.26	Saprobes	Pigmented	Septate	EU673330	DQ377857	[11,19]
B. tectonae	MFLUCC 12-0381	Saprobes	Pigmented	Septate	KJ556515	MH878606	[33,118]
B. thailandica	MFLUCC 14-1190	Saprobes	Pigmented	Septate	KY115675	N/A	[119]
Botryobambusa fusicoccum	MFLUCC 11-0143	Saprobes	Hyaline	Aseptate	JX646792	JX646809	[11]
B. fusicoccum	MFLUCC 11-0657	Saprobes	Hyaline	Aseptate	JX646793	JX646810	[11]
Botryosphaeria agaves	MFLUCC 11-0125	Pathogen/ saprobes	No asexual morph recorded		JX646791	JX646808	[11]
B. agaves	MFLUCC 10-0051	Pathogen/ saprobes	No asexual morph recorded		JX646790	JX646807	[11]
B. corticis	CBS 119047	Pathogens	Hyaline	Aseptate	DQ299245	EU673244	[19]
B. dothidea	CBS 115476	Saprobes/ pathogens/ endophytes	Hyaline	Aseptate	AY236949	DQ377852	[11,24,120,121]
B. dothidea	CMW 25413	Saprobes/	Hyaline	Aseptate	KF766167	KF766332	[111,122,123]

		endophytes					
B. dothidea	GZCC 16-0013*	Saprobes	Hyaline	Septate	KX447675	N/A	[124]
B. dothidea	CGMCC 3.17722*	Saprobes/ pathogens	Hyaline	Septate	KT343254	N/A	[125,126]
B. dothidea	CGMCC3.18745	Pathogens	Hyaline	Septate	KX278003	MF410045	[127]
B. fabicerciana	CBS 127193*	Saprobes/ pathogens	Hyaline	Aseptate	HQ332197	N/A	[19,128]
B. fabicerciana	MFLUCC 10-0098	Pathogens/ saprobes/ endophytes	Hyaline	Aseptate	JX646789	JX646806	[11,129]
B. kuwatsukai	PG259	Pathogens	Hyaline	Septate	KJ433390	N/A	[130]
B. puerensis	CSF6052	Pathogens	Hyaline	Aseptate	MT028569	MT029138	[131]
B. qingyuanensis	CERC 2946	Pathogens	Hyaline	Aseptate	KX278000	MF410042	[132]
B. ramosa	CBS 122069*	Saprobes/ pathogens	Hyaline	Aseptate	EU144055	N/A	[19,133,134]
B. ramosa	CGMCC3.18739*	Pathogens	Hyaline	Aseptate	KX277989	MF410031	[132]
B. scharifii	CBS 124703*	Pathogens	Hyaline	Aseptate	JQ772020	N/A	[19]
Cophinforma atrovirens	MFLUCC 11-0425	Endophytes/ pathogens	Hyaline	Aseptate	JX646800	JX646817	[19,132]
C. atrovirens	MFLUCC 11-0655	Saprobes	No asexual morph recorded		JX646801	JX646818	[11]
C. mamane	CBS 117444	Endophytes/ pathogens	Hyaline	Aseptate	KF531822	DQ377855	[19,135,136]
Diplodia africana	CBS 120835	Pathogens	Hyaline	Aseptate	EF445343	MH874653	[137]
D. agrifolia	CBS 132777	Pathogens	Hyaline/ pigmented	Aseptate/ septate	MH866050	MH877500	[19]
D. allocellula	CMW:36468	Pathogens/ endophytes	Pigmented	Aseptate	JQ239397	JQ239410	[19,138]
D. arengae	MFLU 17-2769*	Saprobes	No asexual morph recorded		NR_159828	NG_064503	[139]
D. bulgarica	CBS 124254*	Pathogens	Pigmented	Septate	GQ923853	MH874879	[102]
D. corticola	CBS 112549	Pathogens	Pigmented	Septate	AY259100	AY928051	[140]
D. crataegicola	MFLUCC 150905	Saprobes	Pigmented	Aseptate	MF398871	N/A	[141]
D. cupressi	CBS 168.87	Pathogens	Pigmented	Septate	DQ458893	EU673263	[19]
D. eriobotryicola	CBS 140851*	Pathogens	Pigmented	Aseptate	NR_152462	NG_070047	[142]
D. estuarina	CMW 41231*	Endophytes	Pigmented	Aseptate	NR_152461	N/A	[143]
D. fraxini	CBS 136010*	Pathogens	Pigmented	Aseptate	KF307700	N/A	[144]
D. galiicola	JZB 3140014	Saprobes	Pigmented	Aseptate	MN757871	N/A	[141]
D. malorum	CBS 124130	Pathogens	Pigmented	Septate	GQ923865	MH874878	[19,145]
D. mutila	CBS 112553	Pathogens/ saprobes/ endophytes	Pigmented	Septate	AY259093	AY928049	[19,90,146]
D. neojuniperi	CPC 22753*	Pathogens/ saprobes	Pigmented	Septate	NR_152460	N/A	[33,106]
D. olivarum	CBS 121887	Pathogens	Hyaline/ Pigmented	Aseptate/ Septate	EU392302	MH874707	[19]
D. pseudoseriata	CBS 124906	Endophytes/ pathogens	Pigmented	Aseptate	EU080927	MH874931	[19,128]

D. pseudoseriata	CBS 124931	Pathogens/ endophytes/ saprobes	Pigmented	Septate	MH863427	MH874935	[19,24,33]
D. quercicola	CFCC 53769*	Pathogens	Hyaline	Aseptate	NR_169975	MN215866	[121]
D. quercivora	BL8	Pathogens	Hyaline/ pigmented	Aseptate/ septate	JX894205	N/A	[147]
D. rosulata	CBS 116470	Pathogens/ endophytes	Pigmented	Septate	EU430265	DQ377896	[19,148,149]
D. sapinea	CBS 393.84*	Pathogens/ endophytes/ saprobes	Pigmented	Aseptate	NR_152452	DQ377893	[32,33,146]
D. sapinea	CBS 124462	Saprobes/ pathogens	Pigmented	Aseptate	GQ923858	MH874896	[149–151]
D. scrobiculata	CBS 109944*	Pathogens/ endophytes	Pigmented	Septate	DQ458899	EU673268	[19,20,152]
D. seriata	CBS 112555	Saprobes/ / endophytes pathogens	Pigmented	Aseptate	AY259094	AY928050	[19,153,154]
D. subglobosa	CBS 124133*	Pathogens	Pigmented	Septate	GQ923856	N/A	[155,156]
D. torilicola	IT3612*	Saprobes	Pigmented	Aseptate	MK625223	N/A	[157]
D. tsugae	CBS 418.64	Pathogens	Hyaline	Aseptate	DQ458888	DQ377867	[19]
Dothiorella acacicola	CPC 26349	Saprobes	Pigmented	Septate	KX228269	KX228320	[15]
D. acericola	KUMCC 18-0137*	Saprobes	Pigmented	Septate	MK359449	N/A	[158]
D. alpina	CGMCC 3.18001	Saprobes/ pathogens	Pigmented	Septate	KX499645	N/A	[159,160]
D. alpina	KUMCC 18–0135	Saprobes/ pathogens	Pigmented	Septate	MT002267	MT002266	[160,161]
D. americana	CBS 128309*	Pathogens	Pigmented	Septate	HQ288218	MH876298	[19]
D. brevicollis	CBS 130411*	Endophytes/ pathogens	Pigmented	Septate	JQ239403	JQ239416	[19,138]
D. capri-amissi	CBS:121763	Pathogens	Pigmented	Aseptate/ septate	EU101323	KX464301	[111]
D. casuarini	CBS 120688*	Pathogens	Pigmented	Septate	DQ846773	MH874647	[19,162]
D. citricola	ICMP16828	Pathogens	Pigmented	Septate	EU673323	N/A	[146]
D. dulcispinae	CMW:36460	Endophytes	Pigmented	Septate	JQ239400	JQ239413	[19]
D. dulcispinae	CMW 25407*	Pathogens/ endophytes	Pigmented	Aseptate or septate	EU101300	KF766329	[111,148]
D. iranica	IRAN1587C*	Pathogens/ saprobes	Pigmented	Septate	KC898231	N/A	[146,163]
D. lampangensis	MFLUCC 18-0232	Saprobes	Pigmented	Septate	MK347758	N/A	[164]
D. longicollis	CBS 122068	Endophytes	Pigmented	Septate	EU144054	MH874718	[19]
D. magnoliae	CFCC 51563	Pathogens	Pigmented	Septate	KY111247	N/A	[165]
D. mangifericola	IRAN1584C*	Pathogens	Pigmented	Septate	KC898221	N/A	[146,165]
D. mangifericola	CBS 121760*	Pathogens	Pigmented	Septate	EU101290	N/A	[19]
D. moneti	MUCC505	Endophytes	Pigmented	Septate	EF591920	EF591937	[19]
D. plurivora	IRAN1557C*	Pathogens/ endophytes	Pigmented	Septate	KC898225	N/A	[90,146,151]
D. pretoriensis	CMW:36480	Pathogens/ endophytes	Pigmented	Septate	JQ239405	JQ239418	[19,42]

D. prunicola	CAP187	Pathogens	Pigmented	Septate	EU673313	EU673232	[146]
D. rhamni	MFLUCC 14-0902	Saprobes/ weak pathogen	Pigmented	Septate	KU246381	KU246382	[122,166]
D. rhamni	CBS 140852*	Pathogens	Pigmented	Aseptate	KT240287	MH878199	[142]
D. santali	MUCC 509	Endophytes	Pigmented	Septate	EF591924	EF591941	[19]
D. sarmentorum	IMI 63581b	Saprobes/ pathogens	Pigmented	Septate	AY573212	AY928052	[19,167]
D. sarmentorum (? *D. californica*)	CBS 141587*	Weak pathogens/ endophytes	Pigmented	Septate	KX357188	N/A	[140]
D. sarmentorum (? *D. sempervirentis*)	IRAN1583C*	Pathogens/ saprobes	Pigmented	Septate	KC898236	N/A	[122,146]
D. sarmentorum (? *D. iberica*)	CBS 115041	Saprobes/ pathogens/ endophytes	Pigmented	Septate	AY573202	AY928053	[19,90,168]
D. sarmentorum (? *D. guttulata*)	MFLUCC 17-0242	Saprobes	Pigmented	Septate	KY797637	KY815014	[169]
D. sarmentorum	MFLUCC 17-0951	Saprobes/ Pathogens/ endophytes	Pigmented	Septate	MG828897	N/A	[8,89,121, 170]
D. sarmentorum (? *D. omnivora*)	CBS:392.80	Pathogens/ saprobes	Pigmented	Septate	KX464133	KX464348	[122,171]
D. sarmentorum (? *D. parva*)	IRAN1579C*	Pathogens/ endophytes	Pigmented	Septate	KC898234	N/A	[146,172]
D. sarmentorum (? *D. symphoricarposicola*)	MFLUCC 13-0498	Saprobes/ pathogens	Pigmented	Septate	KJ742379	N/A	[167,168]
D. sarmentorum (? *D. vidmadera*)	CBS:725.79	Saprobes/ weak pathogens	Pigmented	Septate	KX464130	KX464340	[163,166]
D. striata	ICMP 16819	Pathogens	Pigmented	Septate	EU673320	N/A	[146]
D. striata	DAR80992	Pathogens	Pigmented	Septate	KJ573643	N/A	[173]
D. tectonae	MFLUCC12-0382*	Saprobes	Pigmented	Septate	KM396899	N/A	[174]
D. thailandica	MFLUCC 11-0438	Saprobes	Pigmented	Septate	NR_111794	NG_042725	[19]
D. thripsita	BRIP 51876	Saprobes	Pigmented	Septate	KJ573642	N/A	[19]
D. uruguayensis	CBS 124908	Endophytes	Pigmented	Septate	EU080923	MH874932	[19]
D. vinea-gemmae	B116-3	Pathogens	Pigmented	Septate	KJ573644	N/A	[173]
D. viticola	CBS 117009	Pathogens/ endophytes	Pigmented	Septate	AY905554	DQ377873	[175,176]
D. viticola	WA10NO01	Pathogens	Pigmented	Septate	HM009376	N/A	[173]
D. yunnana	CGMCC 3.18000	Saprobes/ pathogens	Pigmented	Septate	KX499644	N/A	[90,159]
Endomelanconiopsis endophytica	CBS 120397	Endophytes/ pathogens	Pigmented	Aseptate	EU683656	EU683629	[177,178]
E. microspora	CBS 353.97	Endophytes	Pigmented	Aseptate	EU683655	EU683628	[19,179]
Eutiarosporella africana	CBS 133854	Endophytes	Hyaline	Aseptate	KC769956	KC769990	[180]
E. dactylidis	MFLUCC 13-0276	Saprobes	Hyaline	Aseptate	KM978944	KM978949	[166,181]

E. darliae	CBS:118530	Pathogens	Hyaline	Aseptate	KX464131	KX464346	[182]
E. pseudodarliae	DAR 82489	Pathogens	Hyaline	Aseptate	KP309796	N/A	[182]
E. tritici	CBS 118719	Pathogens	Hyaline	Aseptate	KF531830	DQ377941	[183]
E. tritici-australis	DAR 82485	Pathogens	Hyaline	Aseptate	KP309788	N/A	[182]
E. urbis-rosarum	CMW:36477*	Endophytes	Hyaline	Aseptate	NR_111705	JQ239420	[184]
Lasiodiplodia aquilariae	CGMCC 3.18471	Endophytes	Pigmented	Septate	KY783442	N/A	[185]
L. avicenniae	CBS 139670*	Endophytes	Pigmented	Septate	KP860835	N/A	[143]
L. avicenniarum	C376	Saprobes	Pigmented	Septate	MK347777	MK347994	[164]
L. brasiliensis	CERC 2284	Pathogens	Pigmented	Septate	KX278010	MF410054	[164,186]
L. bruguierae	CMW42480	Endophytes	Pigmented	Septate	KP860832	N/A	[143]
L. chonburiensis	MFLUCC 16-0376*	Saprobes	Hyaline	Aseptate	MH275066	MH260299	[119]
L. cinnamomi	CFCC 51997*	Pathogens	Hyaline	Aseptate	MG866028	N/A	[187]
L. citricola	CBS 124707	Endophytes/ Pathogens	Pigmented	Septate	GU945354	N/A	[19,188]
L. crassispora	CBS 118741	Pathogens/ endophytes	Pigmented	Septate	DQ103550	DQ377901	[19,176]
L. crassispora	CBS 121770*	Pathogens	Pigmented	Aseptate	EU101307	N/A	[111]
L. euphorbicola	CMM3609	Pathogens/ endophytes	Pigmented	Septate	KF234543	N/A	[189,190]
L. gilanensis	CBS 124704	Endophytes/ pathogens	Pigmented	Septate	GU945351	N/A	[19,191]
L. gilanensis	CBS 128311	Pathogens	Pigmented	Septate	HQ288225	N/A	[19,192]
L. gonubiensis	CBS 115812	Endophytes/ Pathogens	Pigmented	Septate	AY639595	DQ377902	[19,193,194]
L. gravistriata	CMM4564*	Pathogens	Pigmented	Septate	KT250949	N/A	[195]
L. hormozganensis	CBS:177.89	Endophytes/ pathogens/ saprobes	Pigmented	Septate	KX464134	KX464349	[19,94,196]
L. iraniensis	CBS 124710	Endophytes/ pathogens/ saprobes	Pigmented	Septate	GU945346	MH874918	[19,94,197]
L. krabiensis	MFLU 17-2617	Saprobes	Hyaline	Aseptate	MN047093	MN017859	[198]
L. laeliocattleyae	CBS 167.28	Endophytes/ pathogens	Pigmented	Septate	KU507487	DQ377892	[199]
L. lignicola	CBS 134112*	Saprobes	Pigmented	Septate	JX646797	N/A	[19]
L. lignicola	CBS 342.78*	Pathogens/ saprobes	Hyaline	Aseptate	KX464140	N/A	[6,8]
L. lignicola	CGMCC 3.18061*	Saprobes/ pathogens	Pigmented	Septate	KX499889	N/A	[200]
L. macrospora	CMM3833	Pathogens	Pigmented	Septate	KF234557	N/A	[189]
L. mahajangana	CBS:559.70	Endophytes/ Pathogens	Pigmented	Septate	KX464137	KX464353	[19,151]
L. mahajangana	IBL352	Pathogens	Pigmented	Septate	KT154759	N/A	[201]
L. mahajangana	CBS 137785*	Pathogens/ endophytes	Pigmented	Septate	KJ638317	N/A	[176,202,203]
L. margaritacea	CBS 122519	Endophytes/ Pathogens	Pigmented	Septate	KT852959	KX464354	[19,204]
L. mediterranea	BL187	Pathogens	Pigmented	Septate	KJ638321	N/A	[202]
L. microconidia	CGMCC 3.18485	Endophytes/	Pigmented	Septate	KY783441	N/A	[45,160]

		pathogens					
L. pandanicola	MFLUCC 16-0265*	Saprobes	Pigmented	Septate	MH275068	MH260301	[119]
L. parva	CBS 494.78	Pathogens/ endophytes	Pigmented	Septate	EF622084	EU673258	[19,42,192]
L. plurivora	CBS 120832	Pathogens	Pigmented	Septate	EF445362	N/A	[137]
L. pontae	IBL12	Pathogens	Pigmented	Septate	KT151794	N/A	[201]
L. pseudotheobromae	CBS 116459	Saprobes/ pathogens/ endophytes	Pigmented	Septate	EF622077	N/A	[94,119,164]
L. rubropurpurea	CBS 118740*	Pathogens/ endophytes	Pigmented	Septate	DQ103553	DQ377903	[19,42]
L. subglobosa	CMM3872	Pathogens	Pigmented	Septate	KF234558	N/A	[189]
L. syzygii	GUCC 9719.2	Pathogens/ saprobes	Pigmented	Septate	MW081991	MW081988	[8,205]
L. thailandica	CPC 22795	Endophytes/ pathogens/ saprobes	Pigmented	Aseptate	KJ193637	N/A	[94,200,206]
L. thailandica	MFLUCC 18-0244*	Saprobes	Pigmented	Septate	MK347789	NG_066319	[164]
L. theobromae	CBS 164.96	Saprobes/ pathogens/ endophytes	Pigmented	Septate	AY640255	EU673253	[11,19,164, 207]
L. tropica	CGMCC 3.18477	Endophytes	Pigmented	Septate	KY783454	N/A	[185]
L. venezuelensis	CBS 118739*	Endophytes	Pigmented	Septate	DQ103547	DQ377904	[19]
L. viticola	CBS 128313	Pathogens/ endophytes	Pigmented	Septate	HQ288227	NG_058231	[19,192,208]
L. vitis	CBS 124060*	Pathogens	Pigmented	Septate	KX464148	KX464367	[6]
Macrophomina euphorbiicola	CMM 4045	Pathogens	No morphology observed		KU058928	N/A	[209]
M. phaseolina	CBS 227.33	Endophytes/ pathogens	Pigmented	Aseptate	KF951627	DQ377906	[26,210]
M. pseudophaseolina	CPC 21417	Pathogens	Pigmented	Aseptate	KF951791	N/A	[210]
M. vaccinii	CGMCC 3.19503	Pathogens	Hyaline	Aseptate	MK687450	N/A	[211]
Marasasiomyces karoo	CBS 118718	Saprobes	Hyaline	Aseptate	KF531828	DQ377939	[134,183]
Mucoharknessia anthoxanthii	MFLUCC 15-0904	Saprobes	Hyaline	Aseptate	NR_148092	KU246379	[166]
M. cortaderiae	CPC 19974	Saprobes	Pigmented	Aseptate	KM108374	KM108401	[183]
Neodeightonia licuriensis	COAD1780	Pathogens	Pigmented	Septate	KP165429	N/A	[212]
N. microspora	MFLUCC 11-0483*	Saprobes	No asexual morph recorded		KU940110	NG_059668	[213]
N. palmicola	MFLUCC 10-0822	Saprobes/ endophytes/ pathogens	Pigmented	Septate	HQ199221	HQ199222	[11,214]
N. phoenicum	CBS 122528	Saprobes/ pathogens	Pigmented	Septate	EU673340	EU673261	[49,215,216]
N. planchoniae	MFLUCC 17-2427*	Saprobes	No asexual morph recorded		NR_163335	MK347972	[164]

N. rattanica	MFLUCC 15-0313	Saprobes	Pigmented	Aseptate	KX646358	KX646353	[116]
N. rattanicola	MFLUCC 15-0319	Saprobes	Pigmented	Aseptate	KX646359	KX646354	[116]
N. subglobosa	CBS 448.91	Saprobes/ pathogens/ endophytes	Pigmented	Septate	EU673337	DQ377866	[11,160,217]
Neofusicoccum arbuti	CBS 117453*	Pathogens	Hyaline	Aseptate/ septate	AY693976	DQ377914	[19]
N. arbuti	CBS 116131*	Pathogens/ endophytes	Pigmented	Septate	AY819720	DQ377915	[19,218,219]
N. australe	CMW 6837	Pathogens/ endophytes	Hyaline	Aseptate	AY339262	KF766367	[19,193,219]
N. brasiliense	CMM1338	Pathogens	Hyaline	Aseptate	JX513630	N/A	[220]
N. buxi	CBS 116.75*	Pathogens	Hyaline	Aseptate	NR_147366	NG_058256	[6]
N. cordaticola	CBS 123634*	Endophytes	Hyaline	Aseptate	EU821898	MH874849	[19]
N. cryptoaustrale	CMW 23785*	Endophytes/ pathogens	Pigmented	Septate	FJ752742	N/A	[110,221]
N. dianense	CSF6075	Pathogens	Hyaline	Aseptate	MT028605	MT029159	[131]
N. eucalypticola	CBS 115679*	Pathogens/ endophytes	Hyaline	Aseptate	AY615141	KF766368	[19,42,222]
N. eucalyptorum	CBS 115791	Pathogens/ endophytes	Hyaline	Aseptate	AF283686	N/A	[19,24,222]
N. grevilleae	CBS 129518	Pathogens/ endophytes	Hyaline	Aseptate	JF951137	N/A	[19,223,224]
N. hellenicum	CERC1947	Pathogens	Hyaline	Aseptate	KP217053	N/A	[225]
N. hongkongensis	CERC 2973*	Pathogens	Hyaline	Aseptate	KX278052	MF410096	[132]
N. ilicii	CGMCC 3.18311*	Endophytes	Pigmented	Septate	KY350150	N/A	[226]
N. kwambonambiense	CBS 123639	Pathogens/ endophytes	Hyaline	Aseptate	EU821900	MH874850	[19,227]
N. lumnitzerae	CMW41469	Endophytes	Hyaline	Aseptate	KP860881	N/A	[143]
N. luteum	CBS 562.92	Pathogens/ endophytes	Hyaline	Aseptate	KX464170	N/A	[19,193]
N. luteum	CMW 41365*	Endophytes	Hyaline	Aseptate	KP860859	N/A	[143]
N. macroclavatum	CBS 118223*	Endophytes	Hyaline	Aseptate	DQ093196	KX464436	[19]
N. magniconidium	CSF5876*	Pathogens	Hyaline	Aseptate	MT028612	MT029166	[131]
N. mangiferae	CMW7024	Pathogens/ endophytes	Hyaline	Aseptate	AY615185	N/A	[193,226,228]
N. mediterraneum	CBS 121718*	Pathogens	Hyaline	Aseptate	GU251176	MH874696	[19,229]
N. mediterraneum	CBS 113083*	Pathogens	Hyaline	Aseptate	KX464186	KX464465	[6]
N. mediterraneum	CBS 113089*	Pathogens	Hyaline	Aseptate	KX464199	KX464481	[230]
N. microconidium	CERC 3497*	Pathogens	Hyaline	Aseptate	KX278053	MF410097	[132]
N. ningerense	CSF6028*	Pathogens	No asexual morph recorded		MT028613	MT029167	[131]
N. nonquaesitum	CBS 126655	Pathogens	Hyaline	Septate	MH864187	MH875645	[19]
N. occulatum	CBS 128008*	Endophytes/ Pathogens	Pigmented	Septate	EU301030	MH876179	[19,231]
N. parviconidium	CSF5667*	Pathogens	Hyaline	Aseptate	MT028615	MT029169	[131]
N. parvum	CBS 138823*	Endophytes/ pathogens/ saprobes	Hyaline	Aseptate	AY236943	N/A	[164,219]
N. parvum	CBS 137504*	Pathogens	Hyaline	Aseptate	KJ657702	N/A	[232]

N. parvum	MFLUCC 15-0900*	Saprobes	Pigmented	Septate	KY856755	N/A	[230]
N. pennatisporum	MUCC 510	Endophytes	Hyaline	Aseptate/septate	EF591925	NG_060253	[19]
N. pistaciae	CBS 595.76*	Pathogens	Hyaline	Aseptate	KX464163	MH872782	[6]
N. protearum	CBS 114176*	Pathogens/endophytes	Hyaline	Aseptate	AF452539	N/A	[19,30,227]
N. ribis	CBS 115475	Pathogens/endophytes	Hyaline	Aseptate	AY236935	N/A	[19,193,233]
N. ribis	CBS 124924	Endophytes/pathogens	Hyaline	Aseptate	FJ900607	KX464401	[19,128]
N. ribis	CBS 123645*	Endophytes/pathogens	Hyaline	Aseptate	EU821904	N/A	[19,128]
N. sinense	CGMCC 3.18315*	Saprobes	Hyaline	Septate	KY350148	N/A	[226]
N. sinoeucalypti	CERC 2005*	Pathogens	Hyaline	Aseptate	KX278061	MF410105	[132]
N. stellenboschiana	CBS 110864*	Pathogens/endophytes	Hyaline	Septate	AY343407	NG_058259	[6,8,230]
N. terminaliae	CBS 125264	Endophytes/pathogens	Hyaline	Aseptate/septate	GQ471802	KX464519	[234]
N. ursorum	CMW 24480*	Endophytes/pathogens/saprobes	Hyaline	Aseptate	FJ752746	N/A	[8,110]
N. viticlavatum	CBS 112878*	Pathogens/endophytes	Hyaline	Aseptate	AY343381	MH874474	[19,227,230]
N. vitifusiforme	CBS 110887	Pathogens/endophytes	Hyaline	Aseptate	AY343383	MH874455	[19,138,226]
N. vitifusiforme	CBS 120081*	Pathogens	Pigmented	Septate	DQ923533	MN162190	[235]
N. yunnanense	CSF6142*	Pathogens	Hyaline	Aseptate	MT028667	MT029193	[131]
Neoscytalidium dimidiatum	CBS 499.66	Pathogens	Hyaline/pigmented	Aseptate/septate	KF531820	DQ377925	[236]
N. dimidiatum	CBS 122071	Pathogens/endophytes	Pigmented	Septate	EF585540	KF766374	[18,96, 149, 237]
N. dimidiatum	MFLUCC 12-0533	Saprobes/pathogens	Hyaline	Septate	KU179865	KU179864	[238,239]
Oblongocollomyces variabilis	CBS 121774	Saprobes/endophytes	Pigmented	Septate	NR136994	KX464536	[6,138]
Phaeobotryon aplosporum	CFCC 53774	Pathogens	Pigmented	Aseptate	MN215836	MN215871	[121]
P. cupressi	CBS 124700	Pathogens/endophytes	Hyaline	Aseptate	FJ919672	KX464538	[19,117,239]
P. mamane	CPC 12440	Saprobes	Pigmented	Septate	EU673332	EU673248	[19,49]
P. negundinis	MFLUCC 15-0436	Pathogens/saprobes	Pigmented	Aseptate	KU820970	KU820971	[8,240]
P. rhoinum	CFCC 52450*	Pathogens	Pigmented	Septate	MH133924	MH133941	[105]
P. rhois	CFCC 89662	Saprobes/pathogens	No asexual morph recorded		KM030584	KM030591	[19,105]
P. ulmi	CBS 123.30	Saprobes	Pigmented	Septate	KX464232	DQ377861	[8]
Sakireeta madreeya	CBS 532.76	Saprobes	Hyaline	Aseptate	KC769960	DQ377940	[183]
Sardiniella urbana	CBS 141580	Pathogens	Pigmented	Septate	NR_155806	NG_060007	[221]
Sphaeropsis chromolaenicola	MFLUCC 17-1499*	Saprobes	No asexual morph recorded		NR_168871	MT214460	[107]

S. citrigena	ICMP 16812	Saprobes	Pigmented	Aseptate	EU673328	EU673246	[19]
S. eucalypticola	CBS 133993	Saprobes	No asexual morph recorded		JX646802	JX646819	[19]
S. porosa	STE-U 5132	Pathogens	Pigmented	Septate	AY343379	DQ377894	[19,241]
S. visci	CBS 100163	Pathogens/ saprobes	Pigmented	Aseptate	EU673324	EU754215	[19,183,242]
Tiarosporella corymbiae	CBS 142532	Saprobes	Hyaline	Aseptate	KY979769	KY979824	[243]
T. paludosa	CPC 22701*	Pathogens or endophytes	Hyaline	Aseptate	NR_132907	KM108404	[183]
Melanopsaceae							
Melanops castaneicola	CFCC 52980*	Pathogens/ saprobes	Hyaline	Aseptate	MK203065	MK203069	[187]
M. chinensis	CFCC 52982*	Pathogens/ saprobes	Hyaline	Aseptate	MK203067	MK203071	[187]
M. tulasnei	CBS 118.39	Pathogens	Hyaline	Aseptate	FJ824771	DQ377856	[4]
M. tulasnei	CBS 116805	Saprobes	Hyaline	Aseptate	FJ824769	KF766365	[50]
Phyllostictaceae							
Phyllosticta acaciigena	CPC 28295	Saprobes	Hyaline	Aseptate	KY173433	KY173523	[15,40]
P. bifrenariae	CBS 128855*	Pathogens	Hyaline	Aseptate	NR_137770	NG_057993	[39]
P. brazilianiae	LGMF 330*	Endophytes	Hyaline	Aseptate	JF343572	KF206217	[39]
P. capitalensis	CBS 128856*	Endophytes/ pathogens	Hyaline	Aseptate	JF261465	KF206304	[39,41]
P. carissicola	CPC 25665	Endophytes/ pathogens/ saprobes	Hyaline	Aseptate	KT950849	KT950863	[115]
P. minima	CBS 111635	Pathogens	Hyaline	Aseptate	KF766215	EU754194	[3]
P. parthenocissi	CBS 111645	Pathogens	Hyaline	Aseptate	EU683672	DQ377876	[96]
P. philoprina	CBS 616.72	Endophytes	Pigmented	Aseptate	KF154279	KF766341	[3]
P. podocarpi	CBS 111647	Endophytes/ saprobes	Hyaline	Aseptate	KF766217	KF766383	[3,42]
P. rhizophorae	NCYUCC 19-0352*	Endophytes	Hyaline	Aseptate	MT360030	MT360039	[244]
Pseudofusicoccum adansoniae	CBS 122055	Saprobes/ endophytes/ pathogens	Hyaline	Aseptate	EF585523	MH874715	[119,245,246]
P. adansoniae	CMW 26147	Endophytes/ saprobes	Hyaline	Aseptate	KF766220	KF766386	[119,245]
P. ardesiacum	CBS 122062	Endophytes/ Pathogens	Hyaline	Septate	EU144060	MH874717	[97]
P. artocarpi	B0431	Endophytes	Pigmented	Septate	KM006452	N/A	[106]
P. calophylli	MFLUCC 17-2533*	Saprobes	Hyaline	Aseptate	NR_163339	N/A	[164]
P. kimberleyenses	CMW 26156	Endophytes	Hyaline	Septate	EU144057	KF766388	[19]
P. olivaceum	CMW22637	Endophytes/ pathogens	Hyaline	Aseptate	FJ888462	N/A	[19,128]

P. stromaticum	CBS 117448	Endophyte/ pathogens/ saprobes	Hyaline	Aseptate	AY693974	DQ377931	[19]
P. violaceum	CMW22681	Endophytes/ pathogens	Hyaline	Aseptate	FJ888475	N/A	[19,247]
Planistromellaceae							
Kellermania anomala	CBS 132218	Saprobes/ pathogens	Hyaline	Septate	KF766181	KF766351	[2,248]
K. confusa	CBS 131723	Saprobes	Hyaline	Septate	KF766174	KF766344	[2]
K. crassispora	CBS 131714	Saprobes	No asexual morph recorded		KF766175	KF766345	[2]
K. dasylirionicola	CBS 131720*	Saprobes	Hyaline	Aseptate	NR_165954	NG_042703	[2]
K. dasylirionis	CBS 131715	Saprobes	Hyaline	Septate	KF766177	KF766347	[2]
K. macrospora	CBS 131716	Saprobes	Hyaline	Septate	KF766178	KF766348	[2]
K. micranthae	CBS 131724	Saprobes	Hyaline	Aseptate	NR_120197	NG042706	[2]
K. nolinae	CBS 131717	Saprobes	Hyaline	Septate	KF766180	KF766350	[2]
K. nolinifoliorum	CBS 131718	Saprobes	Hyaline	Septate	JX444861	JX444877	[2]
K. plurilocularis	CBS 131719	Saprobes	Hyaline	Aseptate	KF766181	KF766351	[2]
K. ramaleyae	CBS 131722*	Saprobes	Hyaline	Septate	NR_120201	NG_042710	[2]
K. rostratae	CBS 131721*	Saprobes	Hyaline	Aseptate	NR_120202	NG_042711	[2]
K. uniseptata	CBS 131725	Saprobes	Hyaline	Septate	KF766184	KF766354	[2]
K. yuccifoliorum	CBS 131726	Saprobes/ pathogens	Hyaline	Septate	KF766185	KF766355	[2,249]
K. yuccigena	CBS 131727	Saprobes/ endophytes	Hyaline	Septate	KF766186	KF766356	[2,250]
Umthunziomyces hagahagensis	CPC 29917	Saprobes	Hyaline	Aseptate	KY173472	KY173561	[2]
Saccharataceae							
Pileospora piceae	NB-334-4A	Endophytes	Hyaline	Aseptate	MH144181	MH144184	[251]
Saccharata acaciae	CBS:143167*	Pathogens	Hyaline	Aseptate	NR_156651	NG_058503	[252]
S. banksiae	CPC 27698	Saprobes	Hyaline	Aseptate	KY173449	KY173539	[15]
S. capensis	CBS:122693*	Saprobes	Hyaline	Aseptate	NR_121347	MH874758	[30]
S. daviesiae	CPC 29174	Saprobes	Hyaline	Septate	KY173450	KY173540	[15]
S. eucalypti	CBS 140665	Saprobes	Hyaline	Septate	KT950857	KT950871	[15]
S. eucalyptorum	CPC 29222	Pathogens	Hyaline	Aseptate	KY173451	KY173541	[15]
S. hakeae	CPC 29204	Pathogens	Hyaline	Aseptate	KY173452	KY173542	[15]
S. intermedia	CBS 125546*	Endophytes	Hyaline	Aseptate	NR_156539	NG_057856	[253]
S. kirstenboschensis	CBS 123537	Endophytes	Hyaline	Aseptate	KF766225	FJ372409	[14]
S. lambertiae	CPC 29216	Saprobes	Hyaline	Aseptate/ septate	KY173459	KY173549	[15]
S. leucospermi	CBS:122694*	Saprobes	Hyaline	Aseptate	NR_168142	N/A	[230]
S. petrophiles	CPC 29151	Endophytes	Hyaline	Aseptate	KY173463	KY173553	[15]
S. proteae	CBS 115206	Saprobes/ endophytes/ pathogens	Hyaline	Aseptate	KF766226	DQ377882	[11,14,29, 230]
Septorioides pini-thunbergii	CBS 473.91	Endophytes/ pathogens	Hyaline	Aseptate	KF251243	KF251746	[5,254]
S. strobi	CBS141443	Pathogens	Hyaline	Aseptate/ septate	KT884699	KT884685	[5]
S. strobi	CBS141444	Pathogens	Hyaline	Aseptate/ septate	KT884700	KT884686	[5]

Acrospermales							
Acrospermum longisporium	MFLU 17-2849	Saprobes	No asexual morph recorded		N/A	MG815827	[139]
Asterinales							
Morenoina palmicola	MFLUCC 15-0284	Saprobes	No asexual morph recorded		MK120273	MK120272	[255]
Cladosporiales							
Cladosporium cladosporioides	CBS 127286	Endophytes	Pigmented	Aseptate	MH864505	MH875942	[256]
C. endophyticum	MFLU 18-0005	Endophytes	Hyaline	Aseptate	MG646956	MG646949	[257]
Toxicocladosporium cacti	CBS 141539	Endophytes	Pigmented	Aseptate	NR_152376	NG_069452	[258]
T. immaculatum	CBS 141540	Endophytes	Hyaline	Aseptate	NG_058459	NR_152377	[258]
Dothideales							
Aureobasidium microstictum	CBS 342. 66	Endophytes/ pathogens	Hyaline	Aseptate	FJ150903	FJ150945	[259]
A. proteae	CPC 2824	Endophytes/ pathogens	Hyaline	Aseptate	JN712491	JN712557	[224]
A. pullulans	CBS 109810	Endophytes	Hyaline	Aseptate	FJ150901	FJ150953	[181]
A. pullulans	MFLUCC 14-0288	Endophytes/ saprobes/ pathogens	Hyaline	Aseptate	KM388542	KM461701	[181,260]
Diaporthe arengae	CBS 114979	Endophytes	Hyaline	Aseptate	NR_111843	N/A	[261]
D. pandanicola	MFLUCC 17-0607	Endophytes	No asexual morph recorded		NR_172400	N/A	[257]
Kabatiella bupleuri	CBS 131304	Endophytes/ saprobes	Hyaline/ pigmented	Aseptate/septate	KT693738	N/A	[262]
Sydowia polyspora	CBS 116. 29	Endophytes/ pathogens	Hyaline	Aseptate	MH855019	MH866487	[42,263]
Dyfrolomycetales							
Dyfrolomyces tiomanensis	MFLUCC13-0440	Saprobes	No asexual morph recorded		MK028714	MK026760	[264]
Melomastia fusispora	HKAS 121316	Saprobes	No asexual morph recorded		OK623480	OK623464	[265]
Hysteriales							
Hysterium angustatum	MFLUCC 11-0004	Saprobes	Hyaline	Aseptate	MN608547	MN577416	[266]
Jahnulales							
Manglicola guatemalensis	BCC20156	Saprobes	No asexual morph recorded		JN819283	FJ743448	[267]
Kirschsteiniotheliales							
Taeniolella exilis	CBS122902	Endophytes	Pigmented	Aseptate/ septate	MW883451	KX244968	[268]
Magnaporthales							
Mycoleptodiscus atromaculans	MUCL 34983	Endophytes	Hyaline	Aseptate	MK487736	MK487709	[42,269]
Minutisphaerales							
Minutisphaera aquaticum	MFLU 19–2846	Saprobes	No asexual morph recorded		MN857185	MN857176	[270]
M. fimbriatispora	G155-1	Saprobes	No asexual morph recorded		JX474873	JX474859	[271]

Muyocopronales							
Muyocopron dipterocarpi	MFLU 15-1132	Saprobes	No asexual morph recorded		MT137785	KU726966	[272]
Mycosphaerellales							
Acidomyces acidophilus	CBS 125382	Endophytes	No asexual morph recorded		MH863600	MH875076	[273]
Cercospora beticola	CFSCD 6	Endophytes/ pathogens	Hyaline	Aseptate	MZ398294	N/A	[110,274]
Zasmidium scaevolicola	CBS 127009	Endophytes/ pathogens	Pigmented	Septate	NR_156565	KF251789	[275]
Pleosporales							
Acrocalymma vagum	BRPCR16	Endophytes	Pigmented	Septate	MT658022	OL687556	[276]
Alternaria alternata	SF-001	Endophytes/ saprobes/ pathogens	Pigmented	Septate	MW866522	MZ275253	[42,277]
A. arbusti	CBS 596. 93	Endophytes	Pigmented	Septate	MH862447	MH874095	[278]
A. burnsii	L1	Endophytes/ pathogens	Pigmented	Septate	MZ067801	N/A	[257]
Bambusicola massarinia	MFLUCC 11-0389	Saprobes	Pigmented	Septate	NR_121548	NG_058658	[279]
Bipolaris drechsleri	CBS 136207	Endophytes/ pathogens	Pigmented	Aseptate	MH866089	MH877614	[42,110]
B. zeae	CBS 127735	Endophytes	Pigmented	Aseptate	MH864763	MH876203	[110,280]
Curvularia aeria	CBS 533. 70	Endophytes	Pigmented	Septate	MH859836	MH871614	[281]
C. intermedia	C34	Endophytes	Pigmented	Septate	MK304232	N/A	[110,282]
Edenia gomezpompae	CBS 124106	Endophytes/ pathogens	Hyaline	Aseptate	MH863351	MH874875	[110,253]
Kirschsteiniothelia lignicola	MFLUCC10-0036	Saprobes	Pigmented	Septate	HQ441567	HQ441568	[283]
Lophiostoma corticola	FeC108	Endophytes	No asexual morph recorded		MW446986	N/A	[274]
Massarina pandanicola	MFLUCC 17-0596	Endophytes	Hyaline	Aseptate	MG646958	NG_064488	[257]
Neoplatysporoides aloicola	CPC 24435	Endophytes	Pigmented	Septate	KR476719	KR476754	[284]
Paraconiothyrium babiogorense	CBS 128292	Endophytes	Pigmented	Aseptate/ septate	MH864845	MH876291	[42,285]
Petrakia irregularis	CBS 306. 67	Endophytes	Pigmented	Septate	MH858977	MH870670	[42,286]
Phomatodes nebulosa	CBS 503. 75	Endophytes/ saprobes/ pathogens	Hyaline	Aseptate	GU237875	GU238115	[42,161]
Stagonospora nodorum	CBS 127171	Endophytes/ pathogens	Hyaline	Aseptate	MH864458	MH875898	[42,287]
Strigulales							
Strigula acuticonidiarum	HMAS L0138045	Endophytes	Hyaline	Septate	NR_146254	N/A	[288]
Valsariales							
Myrmaecium fulvopruinatum	CFCC 52769	Pathogens/ Saprobes	Hyaline	Aseptate	MH458908	MH458902	[187]
Venturiales							

Fusicladium betulae	R6	Endophytes	Pigmented	Septate	AY671908	N/A	[289]
Venturia cephalariae	CBS 372.55	Pathogens	No asexual morph recorded		EU035451	N/A	[290]
V. fraxini	CBS 374. 55	Endophytes	Pigmented	Septate/ Aseptate	KC464358	MH869056	[291]
V. orni	CBS 140924	Endophytes	Pigmented	Septate/ Aseptate	NR_170751	NG_070941	[291]
***Tubeufiales* (outgroup)**							
Helicomyces roseus	CBS 283.51	Saprobes/ endophytes	Hyaline	Septate	AY916464	AY856881	[292,293]
Helicosporium guianense	CBS 269.52	Saprobes	Hyaline	Septate	AY916487	AY856893	[292]

"N/A" sequences data are not available, ? : Problematic synonyms.
